# Review on thread profile modification methods for the planetary roller screw mechanism

**DOI:** 10.1016/j.isci.2026.116249

**Published:** 2026-06-08

**Authors:** Yanqiang Sun, Kun Zhang, Zhaoyao Shi, Guiping Xie, Xinyu Lv, Huiming Cheng, Zhengyi Tang

**Affiliations:** 1 School of Construction Machinery, Shandong Jiaotong University, Jinan 250357, China; 2 Beijing Engineering Research Center of Precision Measurement Technology and Instruments, Beijing University of Technology, Beijing 100124, China; 3 Zhejiang Xiaxia Precision Manufacturing Co., Ltd, Ningbo 315202, China; 4 State Key Laboratory of High-performance Precision Manufacturing, Dalian University of Technology, Dalian 116024, China

**Keywords:** applied sciences, engineering

## Abstract

The planetary roller screw mechanism (PRSM) is a high-precision actuator, widely used in aerospace, humanoid robots, and automotive systems. However, inherent structural and loading characteristics lead to uneven load distribution among thread teeth, causing contact stress concentration and accelerated wear, which limit transmission performance. Thread profile modification has emerged as an effective solution to optimize load distribution and reduce stress concentration. This study reviews the origin, classification, load-bearing characteristics, and kinematics of PRSM. It discusses local modification methods (half-thread thickness, pitch diameter, crest, and root) and global strategies (concave-convex arc reconstruction, multi-parameter optimization, etc.), highlighting their mechanisms, advantages, and limitations. Discrete modification can reduce average contact stress by approximately 35.55% and improve load uniformity. Additionally, the study addresses measurement techniques, potential negative effects of modification, and proposes a macro-meso-micro modification framework. Finally, current challenges and future research directions are summarized, providing a theoretical foundation for PRSM engineering applications.

## Introduction

**Figure 1:**
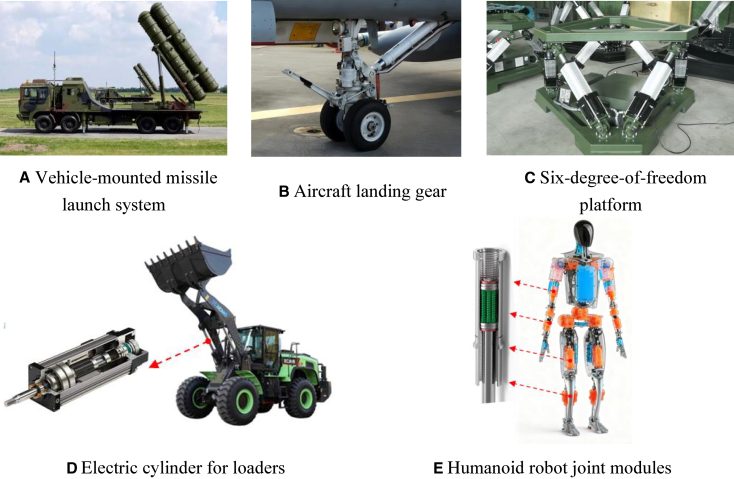
Application fields of PRSM (A) Vehicle-mounted missile launch system. (B) Aircraft landing gear. (C) Six-degree-of-freedom platform. (D) Electric cylinder for loaders. (E) Humanoid robot joint modules.

As a core component of linear actuators, planetary roller screw mechanism (PRSM) is a precision mechanical transmission that converts rotational motion into linear motion. Its primary transmission elements consist of the screw, the nut, and the rollers. In practice, motion and power are transmitted through multi-point threaded engagement formed among the multiple rollers, the screw, and the nut.^1^^,^^2^^,^^3^^,^^4^ Compared with traditional ball screws, the PRSM demonstrates significant performance advantages. Due to the circumferential uniform distribution structure of the rollers and the larger actual contact area, its load-bearing capacity is increased by six times under the same volume conditions, and it saves one-third of the space under the same load. Its lifespan is increased by 14 times, and its operating temperature range, the axial stiffness, and the transmission accuracy are all improved by two times. As a result, PRSM has gradually become the optimal choice for high-performance actuators due to its superior power density, reliability, and lifespan.^5^ Currently, as shown in Figure 1, PRSM has been widely applied in various fields such as military equipment, aerospace engineering, automated production lines, construction machinery, automotive brake-by-wire systems, humanoid robot joint modules, and so on.^6^^,^^7^

In practical applications, the transmission performance of the PRSM is influenced by a complex interplay of factors, including structural design parameters, manufacturing and assembly errors, material mechanical properties.^8^ Among these issues, the non-uniform load distribution among thread teeth, which is caused by the simultaneous engagement of the screw, rollers, and nut, is particularly prominent. This non-uniformity is often characterized by a significantly higher load borne by the first engaged tooth near the loaded end, which triggers localized contact stress concentration, accelerating surface wear and contact fatigue damage. Consequently, these effects restrict the load-bearing capacity and diminish the lifespan of the PRSM.^9^ To address these issues, thread profile modification has emerged as an effective structural optimization strategy. This method involves precise adjustments to the thread profile or geometric parameters, thereby actively modulating both the contact conditions and the stiffness distribution across the engaging teeth. Ultimately, achieving a uniform load distribution enhances the overall transmission performance, operational reliability, and lifespan of the PRSM.

The origin and evolution of the PRSM are first reviewed. Subsequently, typical structural types and their load-bearing characteristics are analyzed systematically. Based on this foundation, the current research status of thread profile modification methods is summarized. The investigation focuses on the underlying mechanisms, advantages, limitations, and engineering applicability of these methods. Finally, the primary issues and technical challenges in existing research are identified and perspectives on future development directions are also provided.

## Origins and evolution of the PRSM

**Figure 2:**
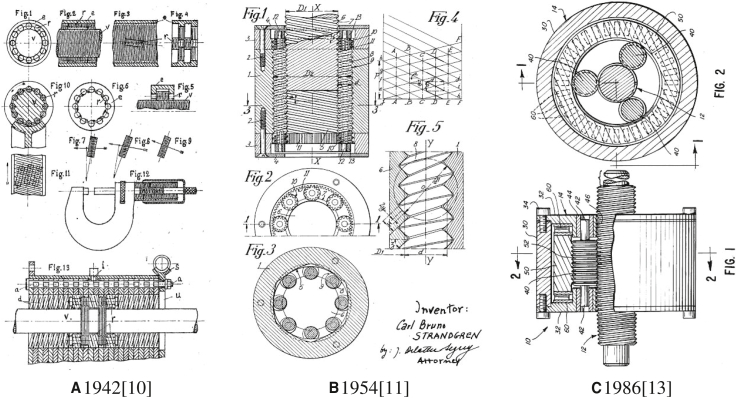
Development of the PRSM (A) 1942.^10^ (B) 1954.^11^ (C) 1986.^13^

The concept of the PRSM dates back to 1942. Carl Bruno Strandgren first proposed a recirculating PRSM structure that was characterized by rollers without a thread lead angle,^10^ as shown in Figure 2A. Over the following decade, Strandgren continued researching this transmission form, introducing both the standard and inverted PRSM configurations. Furthermore, he systematically elaborated on kinematic mechanisms, structural parameter matching, and thread profile design, as illustrated in Figure 2B. These contributions established the theoretical foundation for the industrial application of the PRSM.^11^^,^^12^ Specifically, in the historical patent illustrations shown in Figures 2A and 2B, the labels “v,” “r,” and “e” denote the screw, rollers, and nut, respectively. These markers identify the fundamental interaction between the three primary elements of the mechanism. The configuration in Figure 2C stems from Oliver Saari’s 1986 innovation of integrating the recirculating PRSM with thrust cylindrical roller bearings to develop the bearing-ring PRSM.^13^ This design demonstrates the integration of thrust bearings to manage substantial axial loads, a design feature that has since become a cornerstone of modern heavy-duty PRSM systems. Subsequently, William W. Carson introduced the differential PRSM in 1987 and used a segmented roller engagement method.^14^ By this time, several typical structural forms of the PRSM had been basically established and have remained in use to this day.

For over two decades after its inception, the industrial application of the PRSM remained limited, primarily due to its structural complexity and associated manufacturing challenges. During this initial stage, research focused primarily on linear motion conversion mechanisms and fundamental structural design. Since the 1970s, the PRSM has gained significant attention.^15^ This shift was driven by advancements in precision manufacturing. Furthermore, demand increased for linear actuators with high load-bearing capacity, high precision, and a long lifespan in the military, machine tool, and petrochemical sectors. During this period, specialized manufacturers such as SKF, Rollvis, GSA, Moog, Exlar, and Nook were established, which significantly accelerated the development of the PRSM. Research priorities shifted toward enhancing transmission accuracy and load-bearing performance. At that time, applications were predominantly concentrated in the military sector. In the 21st century, design and manufacturing technologies matured further. The application of the PRSM expanded into automotive manufacturing, medical devices, and automated equipment, evolving into a critical foundational component for high-end machinery. In 2017, GSA acquired Rollvis to form a comprehensive product portfolio^16^ that encompasses screw diameters from 3.5 to 120 mm and leads from 1 to 50 mm. It also supports three to six thread starts with a maximum dynamic load rating of 4,000 kN. In 2022, the Schaeffler Group acquired Ewellix, merging GSA, Rollvis, and Ewellix into a single entity. The group now commands a market share of nearly 70%, which reflects a highly concentrated industrial landscape.^17^

Compared to international markets, PRSM research in China began relatively late. Academic attention only started to emerge in the 1990s. Early efforts were primarily concentrated in universities and research institutes, including Huazhong University of Science and Technology, Nanjing University of Science and Technology, Northwestern Polytechnical University, Chongqing University, and the Beijing Institute of Precision Mechatronics and Controls. Research centered on kinematic principles and key parameter matching, with prototype development and small-scale trial production also being explored.^18^^,^^19^^,^^20^^,^^21^^,^^22^ With increasing demand for high-end equipment, several companies in China developed PRSM design and manufacturing capabilities, including Shaanxi Hanjiang Machine Tool, Nanjing Line-tech, and Shandong Beste Precision.^23^ However, systematic theoretical research remains insufficient, and key core technologies have not yet been fully mastered. Consequently, a gap exists between PRSMs produced in China and international products, particularly in terms of product consistency, machining accuracy, and overall performance.

**Figure 3:**
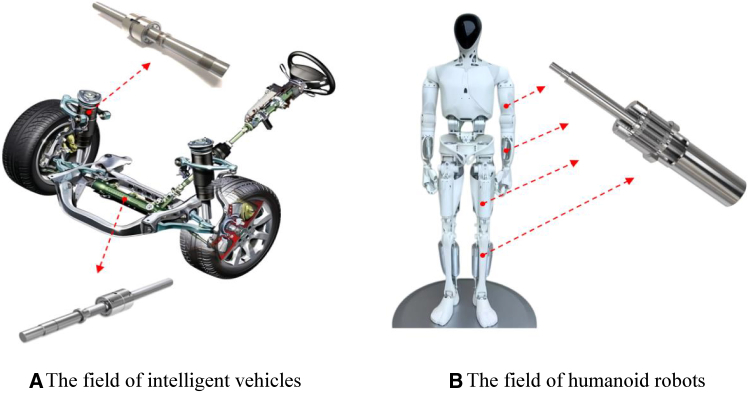
Applications of the PRSM in the fields of intelligent vehicles and humanoid robots (A) The field of intelligent vehicles. (B) The field of humanoid robots.

Since 2023, several strategic policies have been successively released in China. These include the “Robot +” Application Action Implementation Plan and the Guidance on the Innovative Development of Humanoid Robots, along with the Implementation Opinions on Promoting the Innovative Development of Future Industries. Within these frameworks, key technologies and core components of humanoid robots are explicitly designated as priority areas for major breakthroughs.^24^ The inverted PRSM for humanoid robots is entering a new round of development opportunities. High-tech enterprises such as Hangzhou Xinjian, Hubei Kefeng, and Qingdao Guohua are successively investing in research and development and launching new products. As illustrated in Figure 3, PRSM-integrated systems are progressively replacing traditional hydraulic and ball screw solutions in the intelligent vehicle sector, including brake-by-wire, steer-by-wire, and active suspension systems. Such systems achieve millisecond-level response times.^25^ In the field of humanoid robotics, robots like XPENG IRON and Tesla Optimus utilize inverted PRSMs in critical linear joints of their limbs. This configuration enables high thrust output and precise motion control, while also highlighting the PRSM’s superior power density and space utilization efficiency.^26^

## Analysis of classification and load-bearing characteristics of the PRSM

Existing PRSMs are categorized into five basic types, based on the differences in structural configurations and kinematic relationships among the rollers, screw, and nut. These types include standard, inverted, differential, recirculating, and bearing-ring mechanisms, with each type exhibiting distinct differences in structural layout, kinematic characteristics, and engineering applicability. Typical features and corresponding applications are summarized in Table 1.

**Table 1:** Five types of PRSM

No.	Type	Schematic diagram	Action mechanism	Characteristics	Application scenarios
1	Standard		The screw is the driving component. The nut serves as the output component.	Fixed internal ring gears are set inside the nut. Straight teeth at both ends of the rollers engage these gears while the threads mesh.^27^	Large strokes, harsh environments, high loads, and high speeds.
2	Inverted		The nut is the driving component. The screw serves as the output component.	Internal ring gears are removed. External meshing is formed between straight teeth on the screw and the rollers.^28^	Small to medium loads, small strokes, and high speeds. Stroke is limited by thread length.
3	Differential		Motion is transmitted via segmented rollers with varying diameters engaging screw threads and nut ring grooves.	Gear meshing is eliminated. Rollers feature reduced-diameter centers and enlarged ends. This segmented structure meshes with nut bosses and screw threads.^29^	High load-bearing capacity, high stiffness, low wear, and long lifespan. Larger transmission ratios are achieved.
4	Recirculating		Rollers reset to their initial positions after completing one cycle.	Reset cams and cyclic grooves replace the gear mechanisms. This configuration enables periodic cyclic meshing under axial constraints.^30^	High positional accuracy and more meshing points. Higher load capacity is provided, though friction increases.
5	Bearing-ring		The nut rotates freely and transmits power to integrated thrust cylindrical roller bearings.	Thrust bearings are integrated at both ends and fixed with a housing to form the nut. Loads pass from the screw through the rollers to the thrust bearings and housing.^31^	Low friction and high efficiency. Suitable for large axial loads and high axial stiffness.

**Figure 4:**
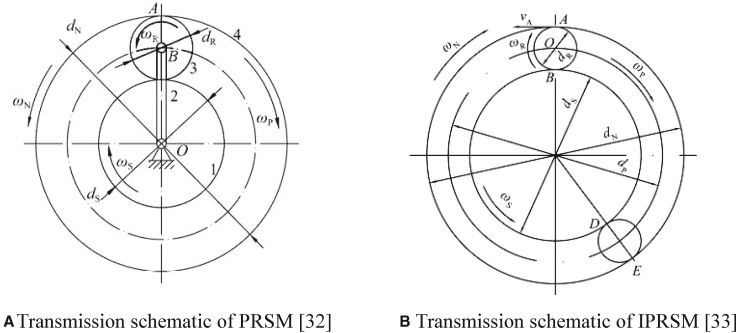
Transmission schematics of two common PRSM types (A) Transmission schematic of PRSM.^32^ (B) Transmission schematic of IPRSM.^33^

All PRSMs convert rotary motion into linear motion based on the planetary transmission principle, and they exist in various forms with different structures and kinematic characteristics. In a standard PRSM, a long screw typically serves as the rotary input. It drives the rollers to rotate around their own axes while revolving along the screw axis. The rollers mesh with the threads of both the screw and an external short nut, and this meshing action pushes the nut to generate axial linear displacement, as shown in Figure 4A.^32^ The inverted planetary roller screw mechanism (IPRSM) adopts an inverted structure with a long nut and a short screw. Generally, a long nut with full-length internal threads acts as the rotary input. The rollers are arranged around the screw and revolve inside the nut, which drives the short screw to achieve long-stroke linear motion, as shown in Figure 4B. Since the outer nut is long, this structure allows a motor rotor to be directly integrated onto its outer diameter. This design effectively improves the axial compactness and power density of the entire system.^33^

In existing research on modification and contact stiffness analysis, the screw, roller, and nut components of PRSM are typically made of high-carbon chromium bearing steel or high-strength alloy steel. These materials are selected for their excellent compression and fatigue resistance. To simulate contact deformation, stress transfer, and micro-yielding behavior under heavy loads, several basic mechanical parameters must be included when building simulation and theoretical models. These parameters mainly include the elastic modulus for resistance to elastic deformation and poisson ratio for lateral deformation effects. Additionally, yield strength is used to define the critical state where the material begins plastic yielding. The inclusion of these key mechanical parameters ensures the accuracy of multi-body contact calculations and nonlinear finite element analysis. It also provides a solid data foundation for evaluating the effectiveness of modification strategies under realistic physical conditions.^34^

Different types of PRSMs exhibit distinct differences in structural configurations, motion transmission modes, and engineering applicability. Standard and inverted PRSMs mainly use gear-ring engagement to achieve synchronous roller motion, which are mature in structure and suit different load and stroke requirements. The differential PRSM eliminates gear mechanisms through segmented meshing. This approach enhances load-bearing capacity and structural stiffness while achieving larger transmission ratios. The recirculating PRSM uses a reset mechanism for periodic cyclic engagement. This design offers high positional accuracy and significant load-carrying potential. The bearing-ring PRSM changes the load transmission path by integrating thrust bearings, which has unique advantages in reducing frictional losses and improving transmission efficiency. These classifications provide a wide range of options for PRSM design and industrial application. They lay a foundation for subsequent performance analysis and research on thread profile modification for different structural forms.

**Figure 5:**
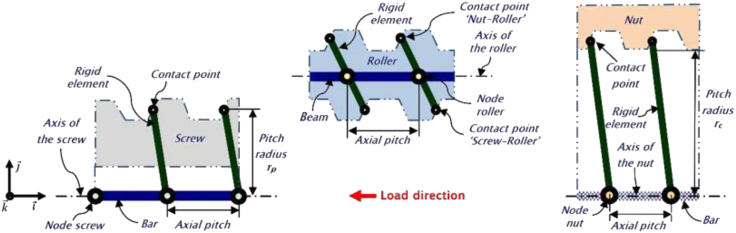
The discrete model of the inverted PRSM^36^

Under high-speed and heavy-load conditions, the load-bearing capacity has become a critical factor limiting the performance enhancement of the PRSM. Due to the intrinsic characteristics of thread transmission, non-uniform load distribution among thread teeth is a common phenomenon for PRSMs under load, where a few front-end thread teeth of a single roller often carry the majority of the load. This leads to localized stress concentration and accelerates wear and fatigue failure. Current research primarily focuses on load distribution patterns within a single roller and methods for achieving uniformity. Meanwhile, load non-uniformity also exists among different rollers. Experimental results indicate that manufacturing errors and assembly deviations cause significant differences in load sharing among rollers.^35^ As shown in Figure 5, some studies assume uniform load distribution among rollers to simplify modeling. In practical applications, this assumption underestimates system stress levels. It also obscures the negative impacts of load non-uniformity on load-bearing capacity degradation, accelerated wear, and shortened lifespan.^36^ To facilitate a more precise evaluation of these mechanical behaviors, discrete analysis models are widely adopted. In the model depicted in Figure 5, the core parameters $r_{p}$ and $r_{c}$ denote the pitch radius of the screw and the pitch radius of the nut, respectively, which define the radial positioning of the meshing nodes. These nodes correspond to discretized mass points for calculating local elastic deformations under load, while the bars represent the structural axial stiffness of individual segments.

**Figure 6:**
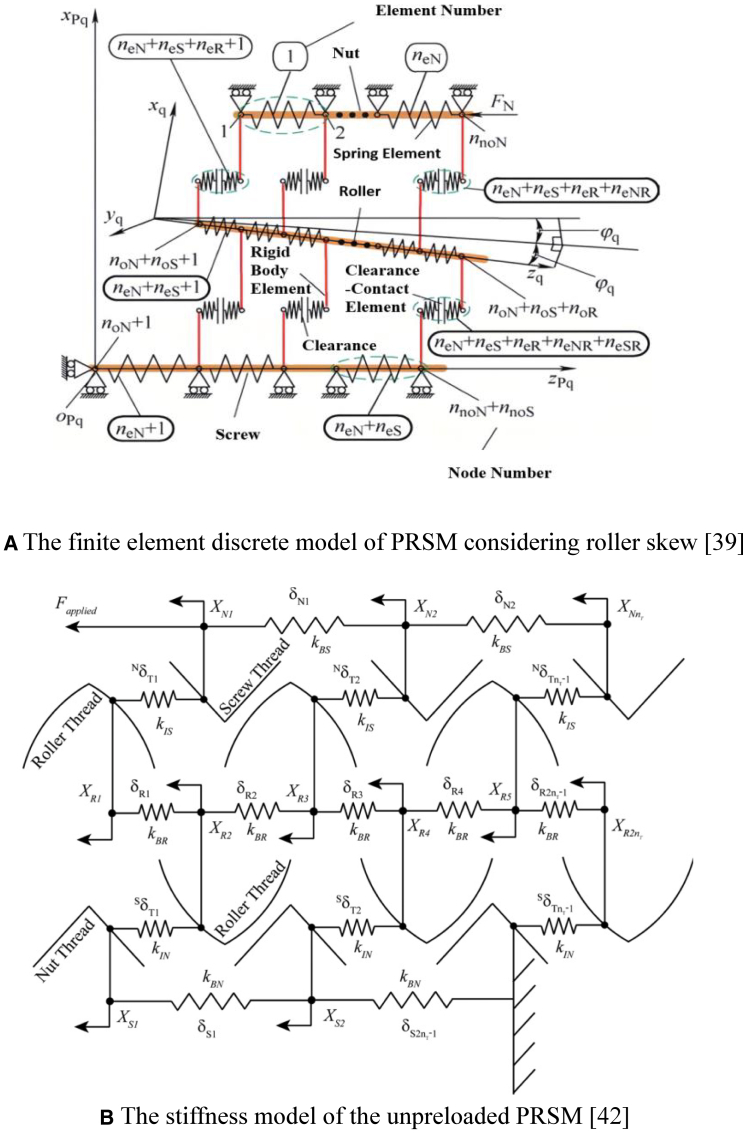
The load distribution computational model of the PRSM (A) The finite element discrete model of PRSM considering roller skew.^39^ (B) The stiffness model of the unpreloaded PRSM.^42^

To address the load distribution of PRSMs, various theoretical and numerical computational models have been developed by scholars both in China and abroad. Yang et al.^37^ systematically analyzed the load distribution characteristics of PRSMs. Their model integrated Hertzian contact deformation, shaft elastic deformation, and thread teeth deformation. Furthermore, experimental data comparison verified the accuracy of their findings. Zhang et al.^38^ combined deformation compatibility with force equilibrium conditions. They further developed a load distribution model that accounts for installation configurations, loading conditions, and thread geometric parameters. Fu et al.^39^ proposed a calculation method for PRSM load distribution that takes into account roller skew and thread disengagement, as illustrated in Figure 6A. A static model was established to consider the skew of rollers relative to the nut and screw, and validation was carried out by comparing its results with finite element models under both error-free and roller-skew conditions. Furthermore, the sensitivity of the PRSM to roller deflection varies significantly depending on the selected thread profile modification scheme. Traditional modification strategies relying on point contact are highly susceptible to assembly errors, where minor skewing can trigger severe localized stress concentration. In contrast, global reconstruction methods, specifically concave-convex arc modification, demonstrate superior robustness and higher error tolerance. By transforming the contact from high-stress point contact to a more favorable near-surface contact state, these schemes effectively increase the actual contact area and distribute the load more evenly across the thread flank, thereby mitigating the negative impacts of assembly-induced roller skewing. Ryś et al.^40^ treated the deformation of rolling elements as that of a rectangular body under shear stress. They established a displacement-force differential equation to determine the load distribution under various installation conditions. Abevi et al.^41^ developed a hybrid model incorporating rod, beam, and nonlinear spring elements. This approach facilitates the efficient and stable evaluation of axial stiffness and load distribution across diverse PRSM configurations. A key advantage of this method is the inclusion of roller bending deformation. Jones et al.^42^ established a global stiffness model for PRSMs based on the direct stiffness method, as shown in Figure 6B, which not only predicts system stiffness but also determines the load distribution of individual thread pairs. This mechanical interaction is further clarified through the parameters visualized in Figure 6. Here, $k_{IS}$, $k_{IR}$, and $k_{\text{IN}}$ denote the equivalent axial stiffness of the screw, roller, and nut threads at each i-th meshing point, respectively. The variable $F_{N}$ represents the total axial load applied to the system. By integrating these spring elements into a deformation compatibility framework, the model provides a rigorous basis for predicting how load is shared among the individual thread teeth.

To address the issue of load distribution in PRSMs under loading conditions, scholars worldwide have made significant progress in the quantitative characterization of load distribution mechanisms. Mechanical models for multi-roller engagement have been established and validated through comparison with experimental tests and finite element analysis.

These findings indicate that these models exhibit high reliability in predicting the global stiffness characteristics of PRSMs and the load distribution of individual rollers, laying a solid foundation for subsequent transmission performance analysis. However, current research still faces limitations. Most models emphasize the passive prediction of load distribution. Under fixed geometric parameters and error conditions, they evaluate the degree of load distribution non-uniformity, but discussions on active design methods for load distribution control remain relatively insufficient. In this context, the method of thread profile modification has attracted increasing attention, which guides the rational allocation of loads across multiple contact surfaces by modifying the thread geometry. It is recognized as an effective way to improve load distribution and enhance load-bearing performance.

PRSM mechanisms most commonly use standard triangular threads with a 90-degree thread angle. The thread profiles of the screw and nut are typically V-shaped and consist of straight lines. In contrast, the roller threads feature single or double arc convex profiles. This design ensures that the thread pairs form stable point or elliptical contact under load.^43^ The geometric modification strategies discussed in this study, such as pitch diameter modification, tip modification, and thread thinning, are summarized from relevant research literature. These methods serve as general geometric tools and are not limited to a specific thread angle. Although some studies use specific angles for their mechanical models, these design ideas can be applied to many different thread structures based on practical engineering needs.

## Research status of thread profile modification methods for PRSM

Thread profile modification methods involve the rational adjustment of local or global thread geometries. These techniques reduce the degree of load concentration and improve load distribution uniformity among thread teeth, thus significantly enhancing the load-bearing capacity, lifespan, and transmission performance of PRSMs. Therefore, on the basis of load distribution research, a systematic review of progress in the PRSM modification methods is of great importance.^44^ This summary helps establish a comprehensive design framework that covers load distribution analysis, modification design, and transmission performance enhancement. At present, major thread profile modification techniques adopted worldwide consist of half-thread thickness modification, pitch diameter modification, tooth tip modification, root modification, concave-convex arc profile modification, parameter optimization, and other specialized modification techniques. We present a comprehensive summary at the end of this chapter, comparing the mechanisms, advantages and limitations of these methods to offer a clear overview of their applicability for the subsequent detailed analysis.

### Half-thread thickness modification

**Figure 7:**
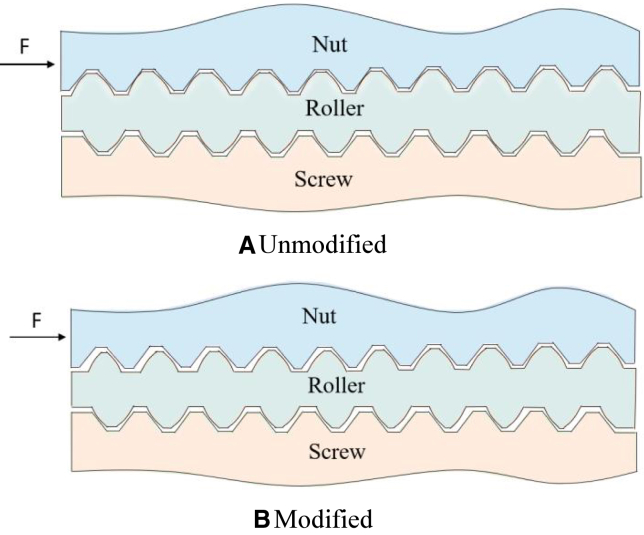
Example of half-thread thickness modification (A) Unmodified. (B) Modified.

Taking the standard-type PRSM as an example, half-thread thickness modification is usually implemented on the premise of maintaining the basic profile of the thread teeth unchanged. This modification is achieved by gradually adjusting the half-thickness of the thread teeth on one or both sides along the axial or helical direction. This modification alters the initial contact state and load transmission path of the thread pair, as shown in Figure 7. Scholars have carried out a variety of modeling and analysis studies for different types of PRSM structures, investigating the influence of half-thread thickness modification on the thread load distribution and contact stress characteristics.

**Figure 8:**
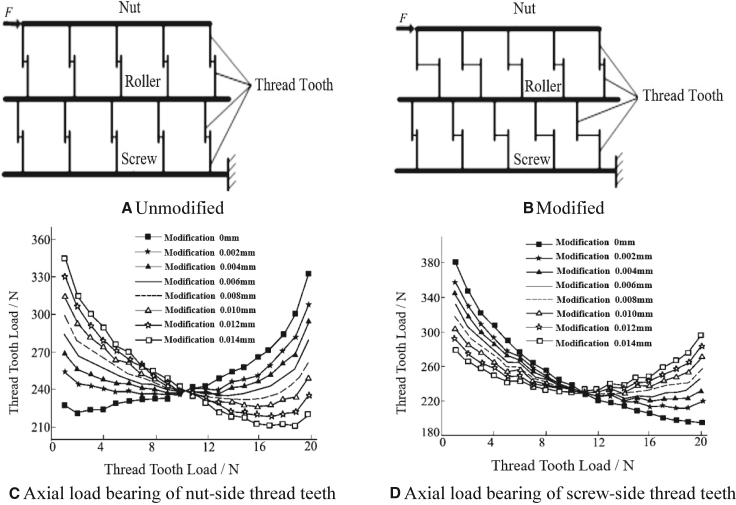
Comparison before and after half-thread thickness modification of roller thread teeth^34^ (A) Unmodified. (B) Modified. (C) Axial load bearing of nut-side thread teeth. (D) Axial load bearing of screw-side thread teeth.

Guo et al.^34^ proposed a half-thread thickness modification method for roller threads based on the relationship between PRSM load distribution and load-induced deformation, as shown in Figures 8A and 8B. The modification amounts on the nut and screw sides were adjusted separately, and their influence on load distribution was analyzed accordingly. Results indicate that this modification method can significantly improve load distribution, and the sensitivity of the load on the two sides to modification amounts differs, as shown in Figures 8C and 8D, the initial load distribution without modification exhibits a sharp peak at the first engaged tooth. After the modification is applied, a distinct “load shifting” effect is observed. For instance, when the modification amount is increased to 0.010 mm, the peak axial load on the nut-side thread teeth is reduced significantly. The load is redistributed to the middle teeth, forming a more balanced “V-shaped” distribution profile. This trend confirms that the optimal modification range can prevent the early fatigue of the first teeth. A reasonable range of modification amounts was determined under specific parameters. This provides a valuable reference for the uniform load design of PRSMs. For differential PRSM structures, Zheng et al.^45^ introduced a modification base and modification coefficients. They designed the thread profiles for the large-diameter segments of the rollers while considering manufacturing feasibility and cost. During the modification process, the axial section profile of the thread was kept unchanged, while the gap between the screw and the roller decreased gradually from the bearing side to the free side. The results show that load allocation is significantly improved through the proper selection of modification coefficients and amounts, and this method enables the uniform load design of differential PRSMs. Regarding inverted PRSMs, Qiao et al.,^46^ based on the circular arc section of the roller thread teeth, performed half-thread thickness modification on the premise of keeping the tooth profile shape unchanged. Through parametric modeling, the half-thread thickness on the screw-roller contact side increased gradually along the helix from the loaded end. On the nut-roller contact side, the thickness decreased gradually from the free end toward the constrained end, with the maximum difference in half-thread thickness serving as the modification indicator. Finite element analysis based on different modification amounts showed that the modification can significantly improve the load uniformity of the thread teeth on both sides. Additionally, the maximum contact stress first decreased and then increased as the modification amount increased.

**Figure 9:**
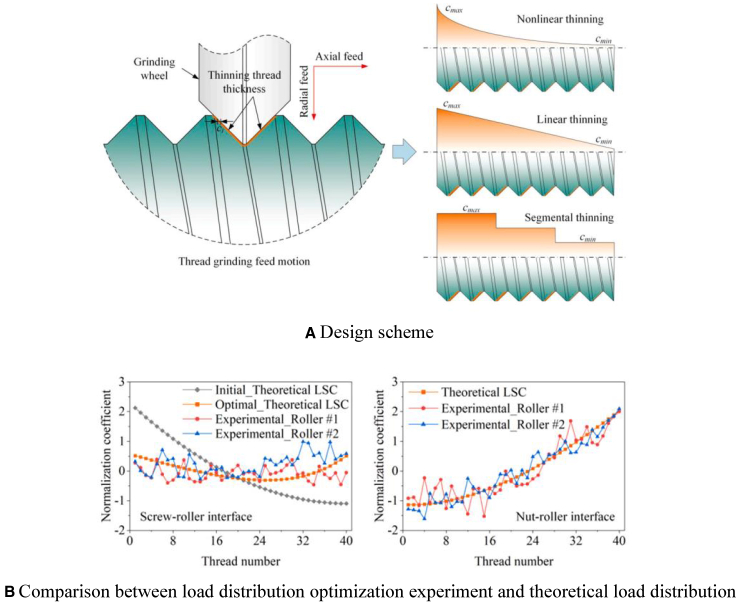
Comparison before and after linear thinning of thread teeth thickness^48^ (A) Design scheme. (B) Comparison between load distribution optimization experiment and theoretical load distribution.

Zhang et al.^47^ established a load distribution model for PRSM threads based on deformation compatibility and force equilibrium. To address the issue of load non-uniformity, a corresponding improvement approach was introduced. This approach compensates for cumulative axial deformation through a redesign of the roller and nut thread geometry, along with an adjustment of the contact conditions with respect to the screw and the nut. The influences of installation methods, loading conditions, and thread geometry were analyzed using a typical PRSM. The results show that the improved design promotes uniform load distribution among the threads. On the basis of a developed PRSM load distribution model, Hu et al.^48^ constructed a multi-objective optimization framework by incorporating the NSGA-II algorithm. Considering manufacturing feasibility and efficiency, they determined a uniform load design scheme featuring linear thinning of thread thickness using the analytic hierarchy process. This method provides a design approach that realizes uniform load distribution through the thinning of thread thickness. The parameter $c_{i}$ denotes the thickness removed from the thread and acts as the core variable for adjusting local contact states. The values $c_{\max}\;\text{and}{\;c}_{\min}$ represent the maximum and minimum thinning amounts respectively, and these two parameters jointly define the variation range of the modification along the thread axis, as shown in Figure 9A. Experimental results indicate that this optimization effectively reduces contact width and its fluctuation range. The approach significantly improves the uniformity of PRSM load distribution and demonstrates its feasibility in load-bearing characteristic tests, as illustrated in Figure 9B.

Half-thread thickness modification provides a feasible approach to improve the load distribution among the thread teeth of a PRSM, which is achieved by adjusting the effective load-bearing capacity of different contact sides along the helical direction. Theoretical analyses and numerical simulations demonstrate the effectiveness of the proposed modification method, which reduces load concentration and improves load uniformity. However, this method mainly regulates the load by changing the local thickness of the thread teeth, so the effective range of the modification is relatively limited. Certain constraints remain regarding its ability to adjust the overall meshing geometry. On this basis, scholars have further focused on influencing the contact state of thread pairs by adjusting global geometric parameters such as the thread pitch diameter. This includes adjusting the thread pitch diameter, which led to the development of the pitch diameter modification method.

### Pitch diameter modification

**Figure 10:**
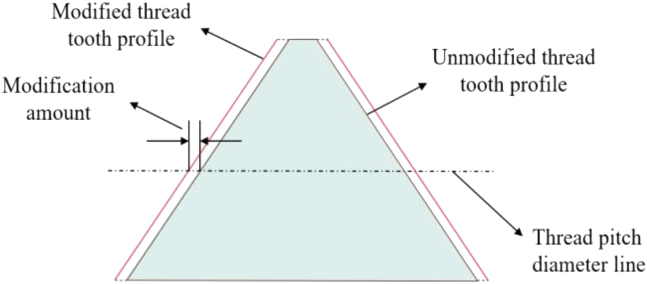
Example of pitch diameter modification

Pitch diameter modification adjusts the pitch diameter dimensions of thread pairs, which influences the contact conditions between the screw and nut pairs and results in a more systematic optimization of load distribution. The thread pitch diameter line serves as a geometric reference standard that provides a baseline for the dimensional changes before and after modification. The amount of pitch diameter modification refers to the change in radial dimensions based on this line, and its value determines the initial gap distribution between thread teeth. By coordinating these parameters, the initial meshing state of the thread pairs under load can be adjusted. This allows the axial load to effectively avoid end regions where stress concentration often occurs, which improves the overall load-bearing stability of the mechanism, as shown in Figure 10.

This method maintains the overall shape of the thread profile while regulating the load-bearing capacity of different thread segments, which is achieved by altering the axial load paths or contact positions during thread engagement. Scholars have conducted theoretical analyses, parameter optimizations, and experimental validations of pitch diameter modification in PRSMs. These studies investigate the effects of pitch diameter modification on load distribution uniformity, contact stress, and overall load-bearing performance.

**Figure 11:**
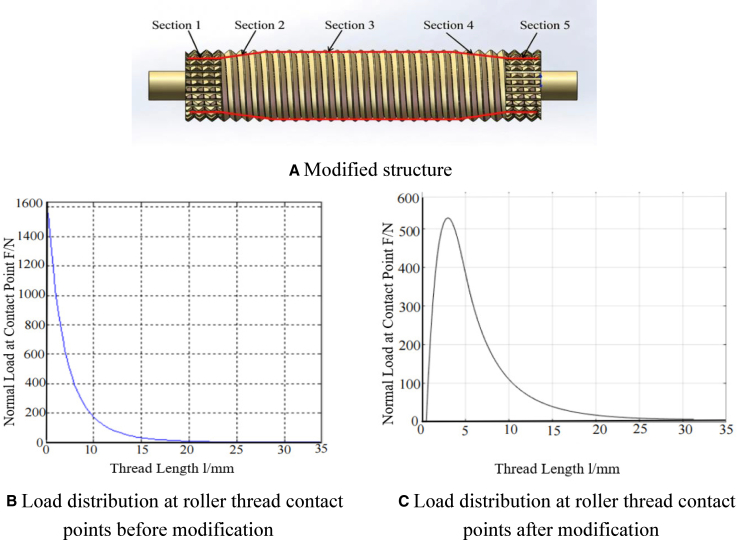
Comparison of segmented rollers before and after pitch diameter modification^49^ (A) Modified structure. (B) Load distribution at roller thread contact points before modification. (C) Load distribution at roller thread contact points after modification.

Zhang et al.^49^ proposed a segmented pitch diameter modification method for roller threads in standard PRSMs. The roller threads were divided into five axial regions, and load regulation was achieved by adjusting the pitch diameter in segments, as shown in Figure 11A. Numerical simulation results indicate that this modification scheme can significantly reduce the normal load on the first few thread pairs at the loaded end, which in turn leads to a more uniform load distribution. As a result, the thread contact state is improved, and the overall load-bearing capacity of the PRSM is enhanced, as illustrated in Figure 11B.

**Figure 12:**
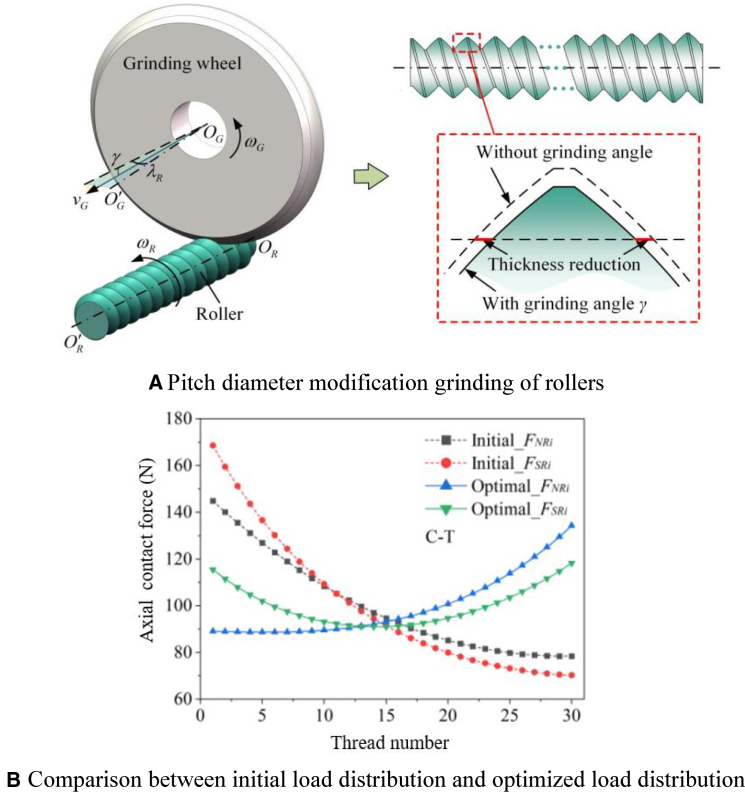
Comparison of rollers before and after pitch diameter modification^50^ (A) Pitch diameter modification grinding of rollers. (B) Comparison between initial load distribution and optimized load distribution.

Hu et al.^50^ proposed an optimization scheme for roller pitch diameter modification, which is based on the principles of deformation compatibility and force equilibrium. Its feasibility was verified through roller grinding experiments, as shown in Figure 12A. Research results indicate that the load distribution factor decreased significantly after the modification, and that the scheme effectively improved load distribution under various external loads and thread counts. These findings confirmed its engineering applicability, as illustrated in Figure 12B.

Pitch diameter modification effectively alleviates load concentration at the first thread teeth and lowers contact stress as well as enhances the overall load-bearing capacity. Its favorable engineering applicability has been demonstrated through numerical simulations and experimental validations. However, pitch diameter modification primarily regulates load distribution by altering global geometric parameters, its ability to control local load peaks remains limited. Meanwhile, the optimization methods for complex structures or multi-condition operating conditions still require further exploration. On this basis, scholars have further proposed the refined regulation of contact characteristics of thread pairs through such means as tooth tip geometry adjustment. This approach provides new research avenues for achieving more uniform load distribution.

### Thread tip modification

**Figure 13:**
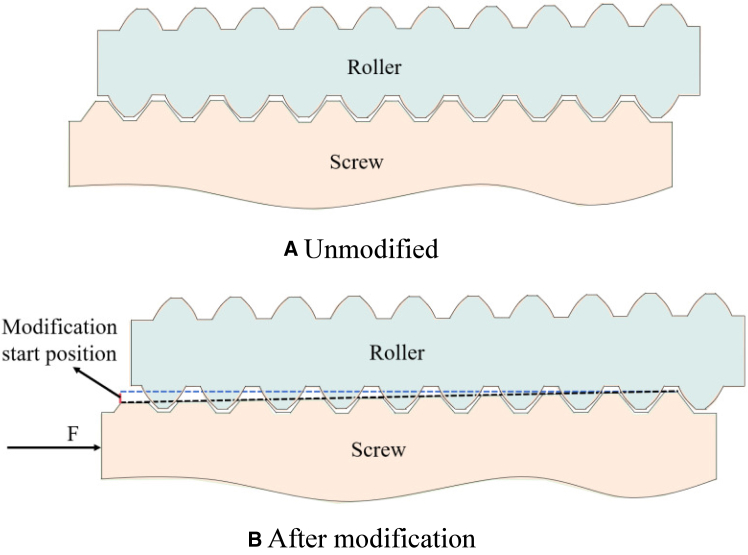
Illustration of thread tip modification (A) Unmodified. (B) After modification.

Taking the inverted PRSM as an example, tooth tip profile modification is usually implemented while maintaining the basic thread profile and pitch diameter parameters unchanged, through a gradual reduction of the thread tip along the axial or helical direction. Such a design provides greater space for elastic deformation during loading, thereby guiding the axial load to transfer gradually backward between thread teeth. The modification start position defines where the modification begins on the flank surface and determines the coverage length of the modification curve. The tip modification depth represents the radial displacement removed from the original tooth tip toward the root. These parameters collectively determine the amount of tip modification, as shown in Figure 13. Scholars have conducted relevant modeling and numerical analysis studies for different types of PRSM structures.

**Figure 14:**
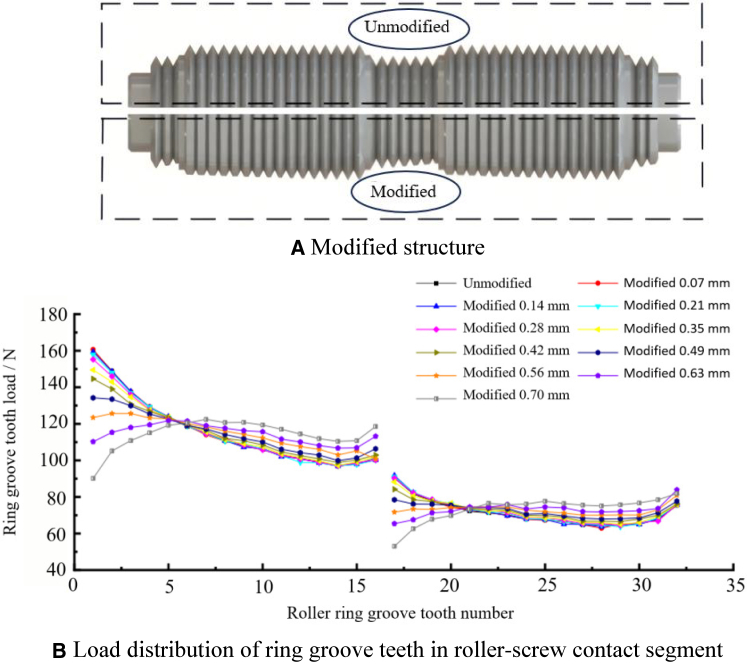
Comparison before and after thread tip modification of roller thread teeth^51^ (A) Modified structure. (B) Load distribution of ring groove teeth in roller-screw contact segment.

Qiao et al.^46^ proposed a tooth tip profile modification method based on the linear cross-section of screw threads, by linearly reducing the height of the meshing thread teeth along the axial direction. This method enables the thread teeth to generate more sufficient elastic deformation during loading, and thus improves the load distribution. Finite element analysis results indicate that appropriate modification promotes load uniformity. However, excessive modification amount will cause some thread teeth to disengage from load-bearing, resulting in increased contact stress and impaired load uniformity. For differential PRSMs, Zhang et al.^51^ proposed a tooth tip profile modification method for roller threads, which causes the height of the thread teeth on the roller that mesh with the screw and nut to decrease linearly along the axis, as shown in Figure 14A. This method improves load distribution by enhancing the deformation capacity of the thread teeth during loading, and the modification amount is characterized by the maximum cutting height of the tooth tip. Finite element analysis results indicate that moderate modification reduces load distribution dispersion and promotes uniform load sharing. Conversely, excessive modification leads to thread disengagement and non-uniform load distribution, as illustrated in Figure 14B. Figure 14B presents a detailed comparison of the load distribution across different ring groove teeth. The data indicate that moderate modification (e.g., 0.28 mm) effectively flattens the load curve. A critical threshold is nevertheless observed at 0.56 mm: beyond this value, the first teeth carry an excessively low load or even disengage, leading to a re-concentration of load on the subsequent teeth. This demonstrates the high sensitivity of the PRSM to the height of tip modification. Additionally, some studies have proposed linear, K-type, and anti-K-type tooth tip modification approaches for the thread teeth of inverted PRSMs. Linear modification involves linear material removal at the thread tips along the direction of the screw force. K-type modification involves simultaneous and equal linear material removal at both the loaded and non-loaded ends of the screw. Anti-K-type modification involves simultaneous and equal linear material removal of tooth height from the middle region of the screw thread toward the loaded end and unloaded end. Finite element analysis results demonstrate that reasonable modification amounts can reduce the contact stress on the first tooth.

Tooth tip modification of thread teeth regulates the load stiffness of thread teeth by reducing their height. This modification approach provides a method based on elastic deformation control to improve PRSM load distribution. Existing research demonstrates that appropriate tooth tip modification effectively alleviates load concentration at the first meshing tooth and promotes progressive load sharing among multiple teeth. However, this method is highly sensitive to the modification amount. Insufficient tooth tip modification yields limited load-leveling effects, while excessive modification tends to cause disengagement of some thread teeth, thus triggering load re-concentration and even an increase in contact stress. Therefore, further research must comprehensively consider contact characteristics and geometric constraints to explore more robust modification strategies. Building on this, scholars have turned their attention to modification methods that adjust thread root geometric features to improve load-bearing conditions.

### Thread root modification

**Figure 15:**
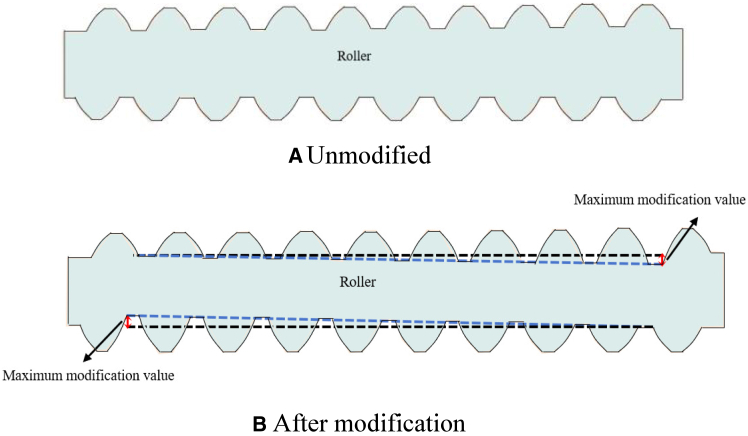
Thread root modification example (A) Unmodified. (B) After modification.

Thread root modification usually involves changing the root depth, chamfer form, or transition curvature. This approach adjusts the equivalent stiffness and stress distribution characteristics of the thread teeth without directly weakening the meshing flank. Consequently, it influences load transfer and distribution among multiple teeth, as shown in Figure 15. Scholars have conducted relevant theoretical analyses and numerical simulations for various PRSM configurations.

**Figure 16:**
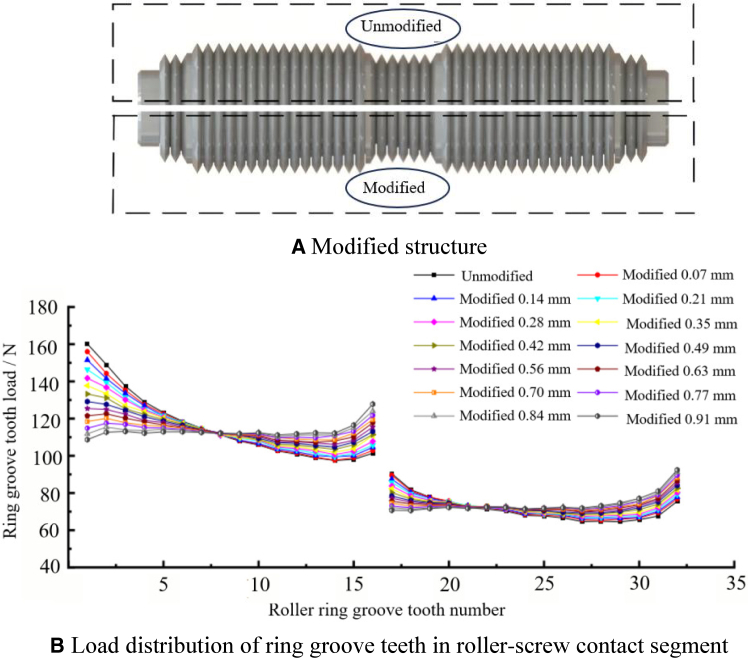
Comparison before and after thread root modification of roller thread teeth^51^ (A) Modified structure. (B) Load distribution of ring groove teeth in roller-screw contact segment.

Zhang et al.^51^ proposed a root-cutting modification method for roller threads. In this method, the root depth in the meshing regions between the roller and both the screw and the nut decreases linearly along the axial direction, thereby extending the equivalent cantilever length of the initial thread teeth. Consequently, their deformation capacity under load is enhanced, which improves the load distribution characteristics, as shown in Figure 16A. The modification amount is characterized by the maximum root-cutting depth, and three-dimensional finite element models under various modification amounts were established to conduct load-bearing performance analysis. Results indicate that the thread teeth load distribution gradually tends to be uniform as the modification amount increases. Under appropriate modification conditions, the dispersion of load distribution is significantly reduced, which achieves an ideal load equalization effect, as illustrated in Figure 16B.

**Figure 17:**
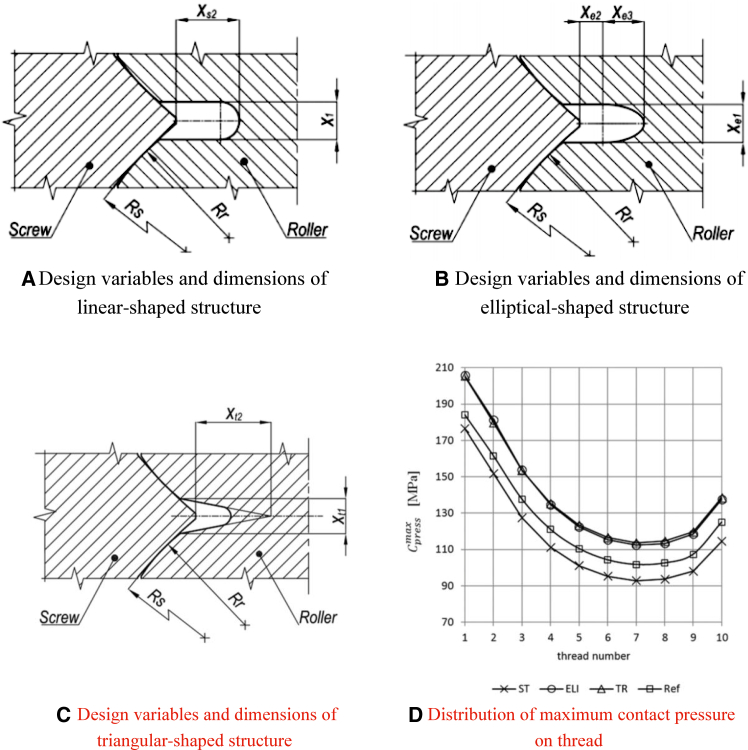
Comparison before and after thread root chamfering of roller thread teeth^52^ (A) Design variables and dimensions of linear-shaped structure. (B) Design variables and dimensions of elliptical-shaped structure. (C) Design variables and dimensions of triangular-shaped structure. (D) Distribution of maximum contact pressure on thread.

Lisowski et al.^52^ investigated the optimal design of roller thread root chamfers for PRSMs based on the finite element method, conducting parameter optimization on chamfer depth, chamfer form, and thread profile curvature radius. The maximum equivalent stress in the chamfer region was adopted as the objective, with contact pressure set as the constraint, as shown in Figures 17A–17C. Optimization results indicate that thread root chamfers effectively alleviate notch stress concentration. Specifically, triangular chamfers demonstrate the superior performance in stress reduction, as illustrated in Figure 17D.

Thread root modification regulates root geometric features and transition forms. While enhancing the structural compliance of thread teeth, this approach effectively alleviates the problem of stress concentration in the root region and exerts a positive influence on improving load distribution among the thread teeth of PRSMs. Existing studies indicate that proper root cutting or chamfer design achieves coordination between load uniformity and local strength without significantly altering the meshing flank geometry. However, the mechanisms of such modification methods rely heavily on local geometric adjustments. Their impact on the global contact state and load transfer path remains relatively limited. On this basis, scholars have further proposed introducing non-linear tooth profiles such as concave-convex arcs. This approach involves the global reconstruction of thread meshing contact geometry, whose goal is to achieve more refined load regulation and contact performance optimization.

### Concave-convex arc modification

**Figure 18:**
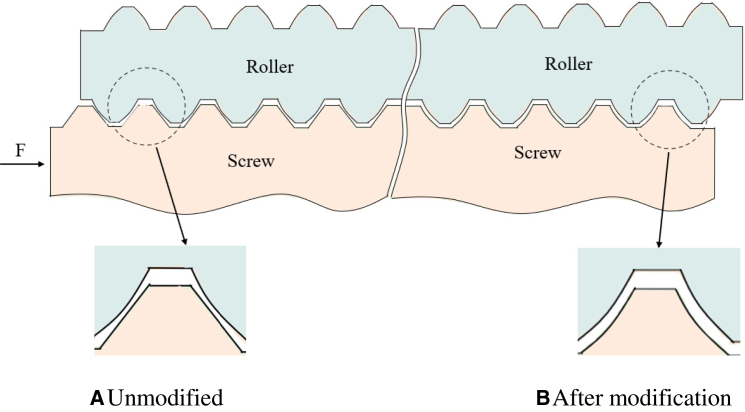
Concave-convex arc modification example (A) Unmodified. (B) After modification.

Taking the inverted PRSM as an example, concave-convex arc profile modification replaces traditional straight or single arc profiles with a combination of convex and concave arcs. This design allows thread pairs to achieve a more favorable surface or near-surface contact state during engagement. Consequently, contact stress is reduced, and the load transfer path is optimized, as shown in Figure 18. Scholars have systematically investigated the load distribution mechanisms and load-bearing performance, which integrate spatial meshing theory, contact mechanics, and numerical optimization methods.

**Figure 19:**
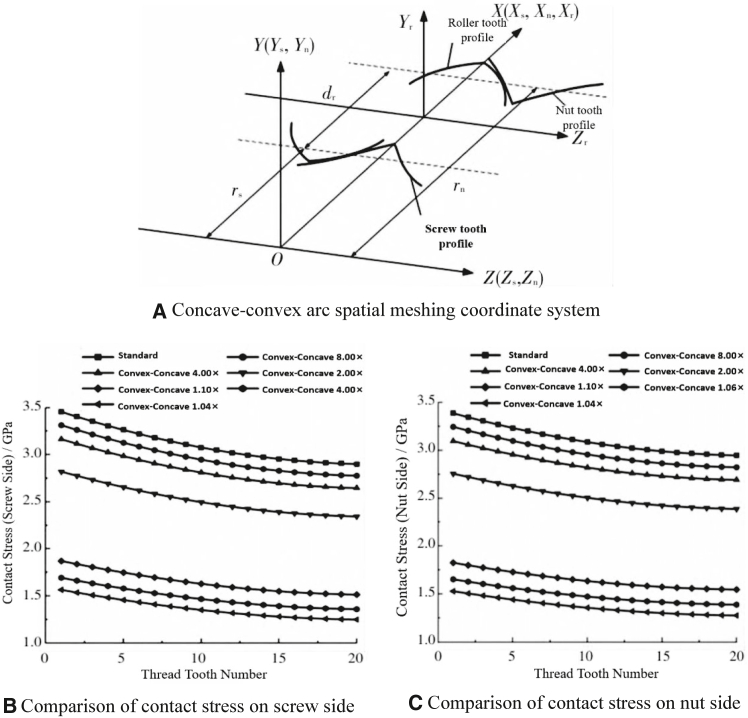
Comparison before and after concave-convex arc modification^53^ (A) Concave-convex arc spatial meshing coordinate system. (B) Comparison of contact stress on screw side. (C) Comparison of contact stress on nut side.

Liu et al.^53^ proposed a PRSM meshing configuration based on concave-convex contact. They designed concave arc thread profiles for the screw and nut, whereby they established a load distribution model integrating spatial meshing theory, Hertzian contact, and deformation compatibility. Numerical methods were used to analyze the contact point position, axial clearance, and contact stress characteristics, as shown in Figure 19A. The results indicate that the concave arc radius exerts a minimal effect on the meshing position but significantly enhances the load-bearing capacity. Compared with the standard PRSM, the concave-convex contact structure significantly improves load-bearing performance. A smaller concave arc radius results in a more pronounced improvement in load-bearing characteristics. The contact stress distribution shown in Figures 19B and 19C reveals that the concave-convex arc modification yields a remarkable stress-leveling effect compared with the standard profile. With a smaller arc radius, the peak contact stress on the screw side is reduced from approximately 3.5 GPa to less than 1.5 GPa. This reduction of more than 50% confirms that the global reconstruction of contact geometry converts the contact state from high-stress point contact to a more favorable distributed contact state, thus significantly extending the theoretical contact fatigue life, as illustrated in Figures 19B and 19C.

**Figure 20:**
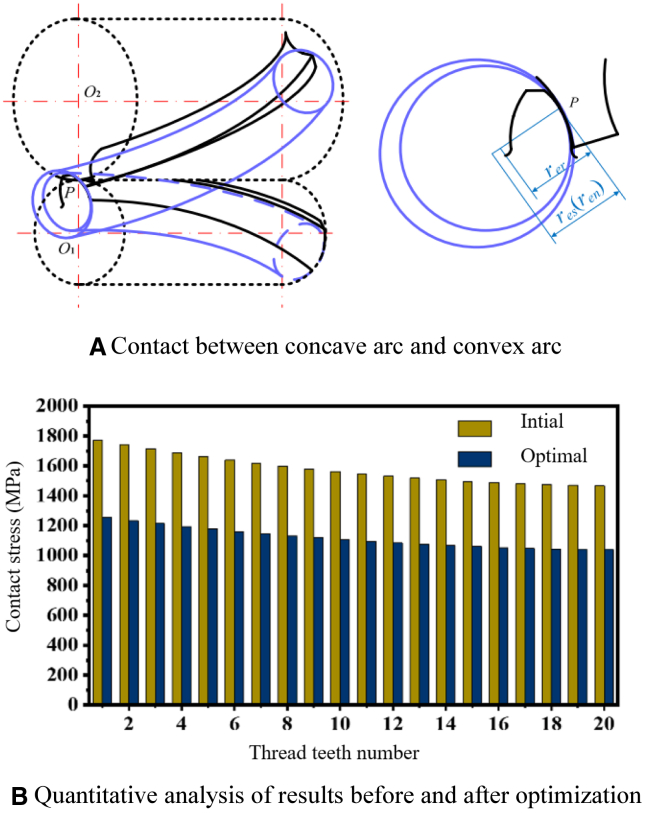
Results of concave-convex arc modification^54^ (A) Contact between concave arc and convex arc. (B) Quantitative analysis of results before and after optimization.

Du et al.^54^ established a PRSM load distribution model based on thread profiles with convex and concave arcs. The maximum contact stresses at both the screw-roller and roller-nut interfaces were incorporated into a multi-objective optimization framework, as shown in Figure 20A. To address the numerous nonlinear constraints introduced by arc-shaped contact, an improved NSGA-II algorithm was adopted to search the complex feasible region. This approach enabled the acquisition of the optimal combination of structural parameters. Results indicate that this multi-objective modeling and optimization method effectively reduces contact stress on thread surfaces. Consequently, the load distribution characteristics of the PRSM are significantly improved, as illustrated in Figure 20B.

Concave-convex arc modification achieves global reconstruction of the thread profile contact geometry, enabling PRSMs to form a more favorable contact state during engagement. This effectively reduces surface contact stress and improves load transfer characteristics. Existing research indicates that this type of modification method offers significant advantages in enhancing load-bearing capacity and optimizing load distribution. It is particularly suitable for applications with high requirements for both heavy load capacity and low stress concentration. Nevertheless, concave-convex arc modification usually involves complex geometric descriptions and nonlinear contact problems. It has a large number of design parameters with strong coupling effects, making the modification outcomes highly sensitive to parameter selection and thus posing certain challenges for practical engineering design and optimization. Building on these considerations, scholars have progressively shifted their focus from isolated geometric modifications toward multi-parameter collaborative regulation. Through systematic parameter optimization methods, comprehensive improvements to modification design have been achieved, thereby fostering the research concept of parameter-optimized modification.

### Parameter-optimization-based modification

Parameter-optimized modification usually takes performance indicators such as load distribution uniformity, contact stress and load-bearing capacity as its optimization objectives. It takes key factors, including thread geometric parameters, meshing clearances, and structural dimensions, as design variables. This approach incorporates diverse methodologies, including genetic algorithms, multi-objective optimization algorithms and finite element analysis, which is designed to seek the optimal combination of modification parameters that satisfies all specified constraints.

**Figure 21:**
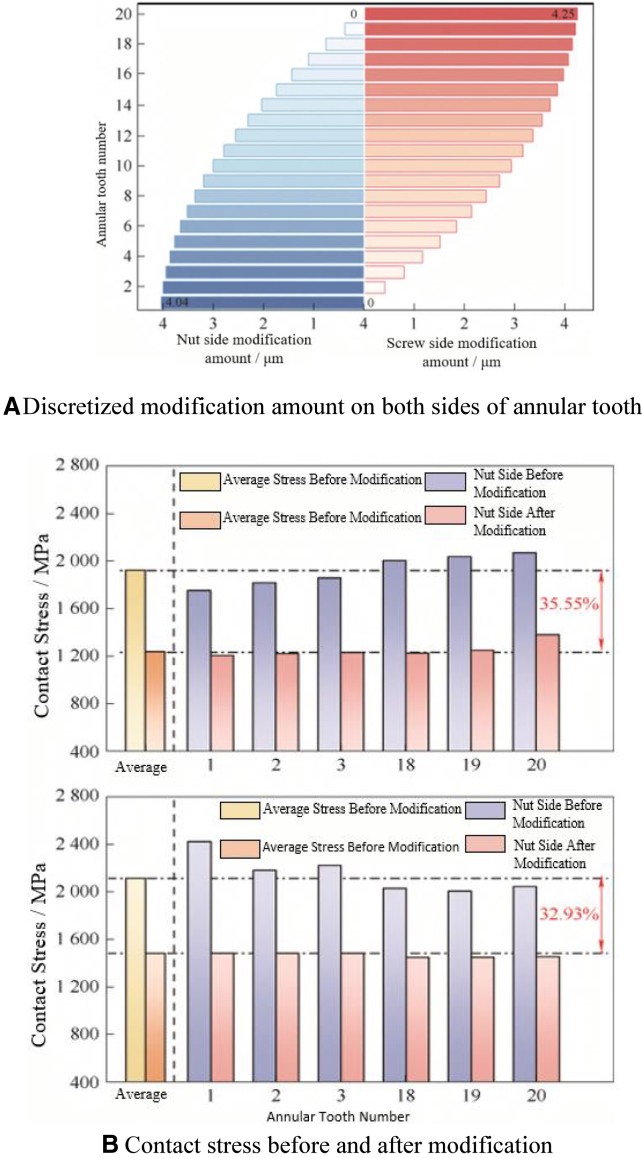
Comparison before and after discretized modification^56^ (A) Discretized modification amount on both sides of annular tooth. (B)Contact stress before and after modification.

Liu et al.^55^ carried out an optimization design for the PRSM load distribution problem based on the MATLAB platform by adopting a genetic algorithm. Key structural parameters with significant impacts on load distribution were selected as optimization variables, while the ultimate load of the thread teeth was applied as a constraint. This approach yielded the optimal parameter combination for the thread load distribution on both sides of the roller. Results demonstrate that the uniformity of the PRSM load distribution is significantly improved after optimization compared to its initial state. Zhang et al.^56^ addressed the problem of insufficient precision in traditional linear compensation modification for recirculating PRSMs by constructing a ring tooth-thread teeth load distribution model. The discrete modification amounts for the roller ring teeth were determined based on deformation coordination and the stiffness matrix, as shown in Figure 21A. Furthermore, a multi-parameter collaborative modification strategy was proposed, accounting for the influences of the thread half-angle and roller profile radius. Finite element results indicate that quadratic polynomial modification facilitates a more uniform load distribution on both the nut and screw sides. Moreover, the average contact stress on both sides of the roller is significantly reduced, confirming the effectiveness of the method, as illustrated in Figure 21B.

**Figure 22:**
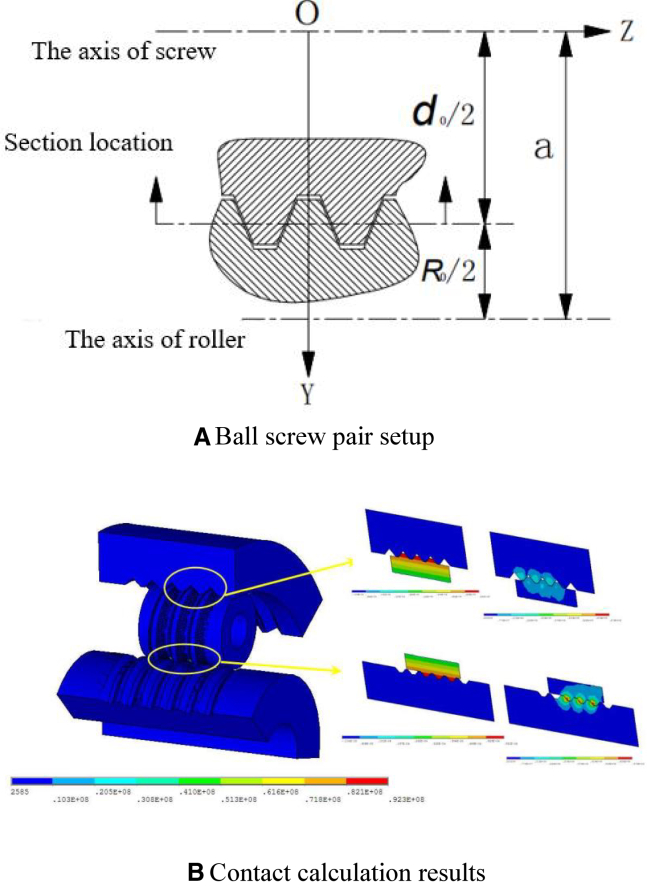
Results of optimized modification^57^ (A) Ball screw pair setup. (B) Contact calculation results.

Based on the meshing principle of PRSMs, Ma et al.^57^ took the pitch-circle tooth thickness as the optimization variable and minimized the meshing clearance of the screw pair while reducing the helix angle. Optimal -structural parameters were obtained through MATLAB-based optimization. Combined with SolidWorks modeling and ANSYS finite element analysis, the contact deformation and stress distribution among the screw, roller, and nut were systematically evaluated, as shown in Figure 22A. Results demonstrate that, under identical lead conditions, PRSMs exhibit lower contact displacement and stress levels owing to a higher number of contact points. Consequently, their meshing stiffness and load-bearing capacity are significantly superior to those of ball screws, as illustrated in Figure 22B.

Parameter optimization modification integrates thread geometric parameters and load distribution performance indicators into a unified optimization framework, facilitating a transition in modification design from empirical adjustment toward systematic optimization. Existing research indicates that this approach effectively improves load distribution uniformity among PRSM thread teeth while enhancing overall load-bearing capacity to a certain extent, provided that structural and load-bearing constraints are satisfied. Nevertheless, parameter-optimization modification relies heavily on the accuracy and computational efficiency of load-distribution models. Furthermore, an increase in the number of optimization variables often incurs significant computational costs. Consequently, its applicability under complex operating conditions and practical engineering applications remains to be further verified. On this basis, scholars have also proposed a number of alternative modification methods tailored to specific structural features or service requirements to supplement and expand the existing modification design approaches.

### Other modification

The techniques mentioned above, including half-thread thickness modification, pitch diameter modification, tooth tip modification, root modification, concave-convex arc profile modification, and parameter optimization modification, cover the mainstream modification methods for PRSMs. There also exist other modification methods for PRSMs that are tailored to specific structural features or performance requirements. These approaches frequently improve the contact states and load transfer characteristics between the rollers, screw, and nut by adjusting thread profile shapes, thread types, or meshing point distributions. Scholars have systematically investigated these modification methods through numerical analysis, finite element simulations, and diverse thread profile designs.

**Figure 23:**
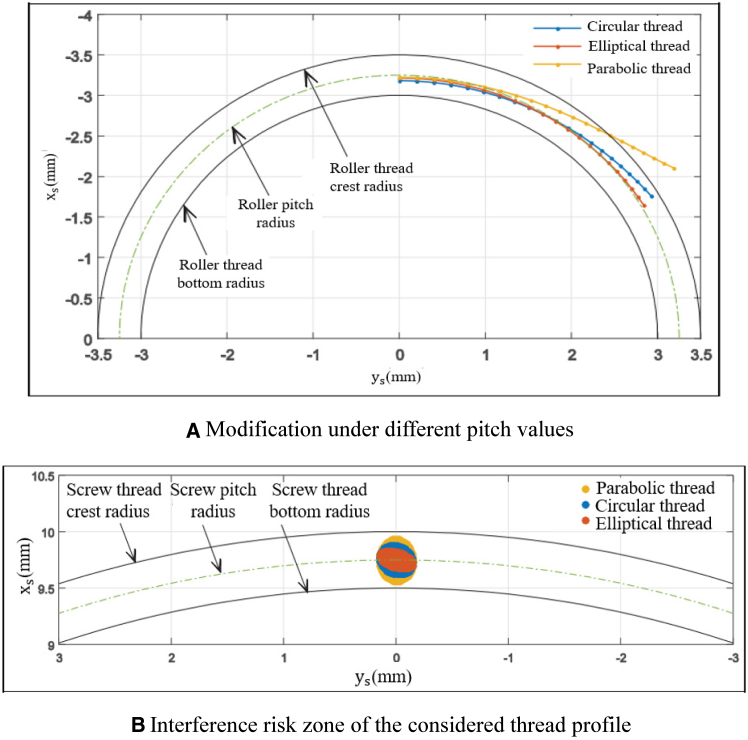
Comparison of circular, elliptical and parabolic modification methods^60^ (A) Modification under different pitch values. (B) Interference risk zone of the considered thread profile.

Zhang et al.^58^ summarized matching methods for key PRSM parameters. In their contact analysis modeling, they approximated the roller arc profile using an equivalent sphere and derived an analytical formula for the axial contact deformation of the PRSM. Based on this, a simplified finite element model was established to conduct contact analysis. The results indicate that the finite element calculations are highly consistent with the theoretical solutions, thereby validating the effectiveness and accuracy of the proposed model and equivalence method. Focusing on the design and analysis of PRSM actuators for high-precision machine tools, Acharya et al.^59^ determined key structural dimensions using analytical methods. They utilized Automatic Dynamic Analysis of Mechanical Systems (ADAMS) and ABAQUS to perform kinematic, dynamic, and stress analyses, comparing the load-bearing characteristics of V-type, trapezoidal, and Whitworth threads. The results demonstrate that trapezoidal threads exhibit lower stress levels and feature mature manufacturing processes, making them more suitable for PRSM structural design. Liu et al.^60^ compared the meshing characteristics of circular, elliptical, and parabolic roller threads in PRSMs. They established mathematical models of the thread surfaces and proposed an analytical calculation method for meshing points, as shown in Figure 23A. The suitability of different thread profiles was evaluated by analyzing the distribution of meshing points and the risk of interference. The results indicate that, compared with traditional circular arc threads, elliptical threads achieve superior meshing positions, which facilitates higher transmission speeds and extends the lifespan, as illustrated in Figure 23B.

Other modification methods provide new perspectives and tools for optimizing PRSM thread teeth load distribution through the diversified design of thread profiles, meshing point distributions, and thread types. Related research indicates that these methods offer distinct advantages in improving load uniformity, reducing contact stress, and enhancing transmission performance. They demonstrate favorable adaptability and engineering feasibility, particularly under specific operating conditions. Nevertheless, these modification techniques often rely on specific structural parameters or application scenarios, which limits their broader applicability and universality. Furthermore, the performance comparisons and optimization patterns among various methods remain incomplete. Based on existing research results, evaluating the effects and limitations of current modification methods and exploring optimization strategies designed in combination with practical engineering needs have become core research priorities. These efforts also serve as a critical direction for advancing the further development and application of PRSMs.

## Comprehensive evaluation and engineering application of PRSM thread modification

### Comparison of different modification methods

A comprehensive comparison is conducted of the six primary PRSM thread teeth modification methods and other modification methods, focusing on their working mechanisms, advantages, and limitations. This comparison allows for a more intuitive assessment of the differences between these methods in improving load distribution, reducing contact stress, and enhancing overall load-bearing capacity. Furthermore, it serves as a practical reference for subsequent modification design and optimization, as shown in Table 2.

**Table 2:** Comparison of different modification methods

No.	Modification method	Mechanism	Advantages and key quantitative results	Limitations and design/manufacturing thresholds
1	Half-thread thickness modification	Adjusts local thread thickness to alter initial contact conditions.	Significant improvement in load uniformity; effectively reduces contact width fluctuations.	Modification range is typically 0.002 mm–0.014 mm; effectiveness diminishes beyond this range.^34^
2	Pitch diameter modification	Adjusts thread pitch diameter to alter axial load transfer paths.	Drastic reduction in normal load on the first tooth (from ∼1,600 N to below 600 N).^49^	Relies on precise grinding angle control; difficult to optimize for multi-condition operations.
3	Tip modification	Adjusts tooth height to guide progressive backward load transfer.	Alleviates load concentration; significantly reduces load distribution dispersion.	Highly sensitive to modification amount; must be kept within 0.70 mm to avoid thread disengagement.^51^
4	Root modification	Changes root geometry or chamfers to regulate equivalent stiffness.	Relieves root stress; triangular chamfers perform best in noise reduction and stress mitigation.	Maximum root-cutting depth is typically around 0.91 mm; limited impact on global load paths.^51^
5	Concave-convex arc profile modification	Reconstructs thread contact geometry to achieve surface or near-surface contact.	Massive reduction in contact stress; can lower stress from ∼3.5 GPa to approximately 1.5 GPa.^53^	Extremely high manufacturing complexity; design parameters are highly sensitive to arc radii.
6	Parameter optimization modification	Utilizes multi-parameter collaborative optimization of thread geometry and load distribution.	Most balanced performance improvement; average contact stress can be reduced by 32.93%–35.55%.^56^	Extremely high precision; high computational cost.
7	Other modification methods	Adjusts profiles and meshing point distributions for specific structures or thread types.	Significant life extension; elliptical profiles offer superior meshing point distribution compared to circular ones.	Lack of universality; manufacturing precision must be tailored to specific models.

Each modification method has a distinct focus on improving PRSM load distribution and contact stress. Half-thread thickness modification, tooth tip modification, and root modification are mainly used for local regulation; they feature simple operation and high engineering feasibility but have a limited ability to regulate the global load path. In contrast, pitch diameter and concave-convex arc profile modifications enable more systematic load regulation and contact optimization, though they demand higher design complexity and present greater manufacturing challenges. Parameter optimization modification facilitates synergistic design through multi-parameter coordination, balancing both localized and global performance. However, it remains highly sensitive to computational accuracy and optimization costs. Alternative modification approaches provide flexible solutions for specialized operating conditions, despite their lack of broad universality. There is also a trade-off between optimizing load distribution and inherent mechanical side effects, as thread modification is a material removal process. This process reduces the effective thickness of the thread teeth and introduces axial physical backlash.^61^ This backlash reduces positioning accuracy because the screw must first rotate to close the gap before the nut starts moving, which causes a direct deviation between the target position and the actual position. Under light loads, material removal causes a mismatch in thread geometry because the thread teeth are locally thinned or cut. The driving thrust is then not enough to maintain full surface contact in the modified area, leading to partial disengagement. This reduces the effective contact area and causes fluctuations in the system’s contact stiffness. During continuous movement, this instability triggers vibration and unstable motion that reduces dynamic transmission accuracy.^62^ Therefore, appropriate modification parameters must be chosen to prevent an unacceptable loss of operating accuracy.

To further promote the engineering application of modification technology, this section discusses the research development and technical roadmap for tooth profile modification. Regarding modification shapes, existing methods often use simple linear or circular profiles. These shapes can cause sudden stress changes at the edges of contact spots under heavy loads. Future research can move toward higher-order continuous surfaces like parabolas. Such smoother profiles would ensure a stable transition of contact pressure and eliminate edge effects. In terms of modification position, asymmetric mechanisms are already used to handle complex conditions. Future efforts should focus on modification techniques under multi-physics coupling. This will allow the layout to be optimized based on wear and load fluctuations over the component’s life. For the calculation of modification amounts, current methods mostly rely on static deformation. However, it is difficult for these to adapt to high-speed alternating loads. A pre-compensation model covering thermodynamic effects could be developed. By including temperature rise and vibration in the initial design, the actual meshing clearance will remain in the best range for bearing loads.

Overall, selecting an appropriate modification method requires considering the structural characteristics, load conditions, and engineering application requirements of PRSMs, or conducting combination and optimization among multiple modification methods to achieve optimal load-bearing performance and lifespan improvement.

The modification methods involved in this study can be classified into two categories: local geometric modification and global geometric modification. Half-thread thickness modification, thread tip modification and thread root modification are mainly realized by adjusting the local geometric dimensions of thread teeth to guide load distribution, which is characterized by local adjustment. Pitch diameter modification and concave-convex arc modification focus on the reconstruction of overall thread meshing parameters or tooth profile geometry, and they fall into the category of global geometric modification. In complex engineering environments, a single modification method often fails to satisfy all performance requirements simultaneously. Consequently, combined strategies following a global-to-local hierarchical adjustment logic are increasingly adopted. Within this framework, global geometric modifications are first employed to rectify the overall load-bearing trend across the entire thread length, while local geometric modifications further mitigate stress concentration at specific heavily loaded teeth. For instance, combining concave-convex arc profiles with multi-parameter optimization achieves the maximum reduction in peak contact stress for aerospace actuators. To ensure these combined strategies work effectively, a systematic optimization process bridges the gap between theoretical design and engineering reliability. High-precision numerical models are established to simulate the meshing contact behavior of parts under load. Sensitivity analysis is then used to identify the critical design parameters that most significantly affect performance. Optimization programs, such as the NSGA-II algorithm, resolve the trade-offs between conflicting performance goals. Finally, the chosen design undergoes rigorous validation on experimental platforms to confirm its effectiveness in actual engineering scenarios.

All these modification technologies are designed to eliminate stress concentration by improving the uniformity of load distribution among thread teeth. The achievement of uniform load distribution is recognized as the core theoretical foundation for further improving the load-bearing capacity, extending the lifespan, optimizing the transmission precision and abating the operational noise of the PRSM.

### Dynamic evolution and operational stability analysis of PRSM

**Figure 24:**
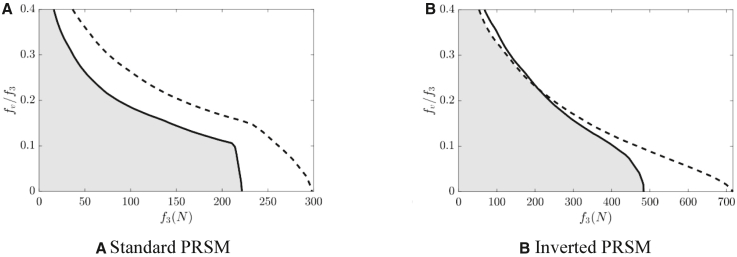
Fatigue life boundary map of PRSM under non-ideal contact distribution^63^ (A) Standard PRSM. (B) Inverted PRSM.

Thread profile modification is a common optimization method in PRSM design, which is usually analyzed based on the initial geometry. However, from initial operation to long-term service, irreversible wear occurs on the thread surface, causing the modified profile to change dynamically. This change is caused by uneven material removal across different areas of the modified thread flanks. Because there is a combined rolling and sliding contact among the screw, rollers, and nut, the modified areas with higher contact pressure or greater relative sliding speed wear much faster. The entire wear process is highly dynamic. As material in the highest contact pressure areas is continuously removed by wear, the contact area and pressure distribution are constantly redistributed, which further changes the location and intensity of subsequent wear.^62^ After long-term operation, the accumulated changes in the thread surface geometry cause the thread shape to gradually deviate from the optimized design state. Consequently, the originally expected uniform load distribution will gradually weaken or even disappear, leading to negative effects such as renewed stress concentration. This phenomenon is especially obvious at the thread ends, which is exactly the area where the stress condition was originally meant to be improved by modification. Ultimately, this causes the actual rolling contact fatigue life to be lower than the theoretically predicted level.^63^ Therefore, to ensure the reliability of the PRSM during long-term operation, it is important to deeply understand the interaction between the wear-induced changes in the thread surface geometry and the load-bearing mechanism. Regarding this phenomenon, Xing et al.^64^ proposed a PRSM wear prediction model based on a general sliding distance. This model reveals the uneven distribution of wear depth in the contact area and confirms that accumulated wear changes the original contact and meshing state over service time. Furthermore, Lepagneul et al.^63^ quantitatively analyzed the impact of non-ideal contact distribution on the rolling contact fatigue life. Their multi-scale modeling results clearly show that an uneven distribution of contact loads causes local threads to bear loads exceeding the system’s infinite life safety range, making the actual contact fatigue life lower than the initial ideal design value, as shown in Figure 24. These studies prove that the modification design needs to include long-term dynamic wear evolution in the life assessment.

**Figure 25:**
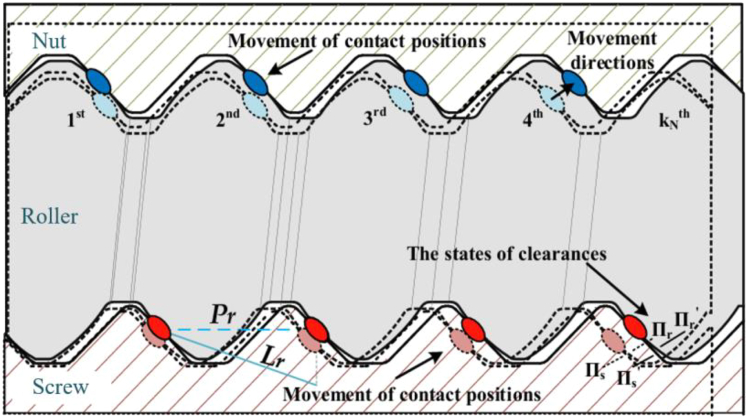
Schematic of thermal deformation in the PRSM components^66^

In addition to surface wear, thermal characteristics also affect the modification performance under high-load and high-speed conditions. The modification depth of the threads is usually only a few micrometers, so even very small differences in thermal expansion among the screw, nut, and rollers can create relative displacements that directly affect the modification gaps. As a result, the designed modification space may be geometrically distorted or even closed, meaning the thread teeth modified to improve load distribution may squeeze each other again due to thermal expansion. This significantly offsets the expected load-sharing benefits and may cause stress concentration to reappear. To ensure reliable operation under high-load and high-speed conditions, the modification design must provide appropriate thermal tolerances to accommodate this expansion rather than relying only on the static geometry. In recent years, researchers have studied the impact of thermal-mechanical coupling on the contact state of the mechanism, and for example, Qiao et al. established a thermal characteristic prediction model for the PRSM based on the thermal network method.^65^ Their experiments and calculations showed that high external loads and high speeds significantly increase internal friction heat, leading to temperature rise. Furthermore, Miao et al. studied contact behavior under thermal-mechanical coupling and pointed out that differences in structures and cooling conditions cause uneven temperature distribution within the system. This local temperature difference leads to micrometer-level thermal deformation between the screw and the rollers, which changes the actual contact position and gap size of the thread pairs and ultimately causes fluctuations in the load-carrying capacity, as shown in Figure 25.^66^

**Figure 26:**
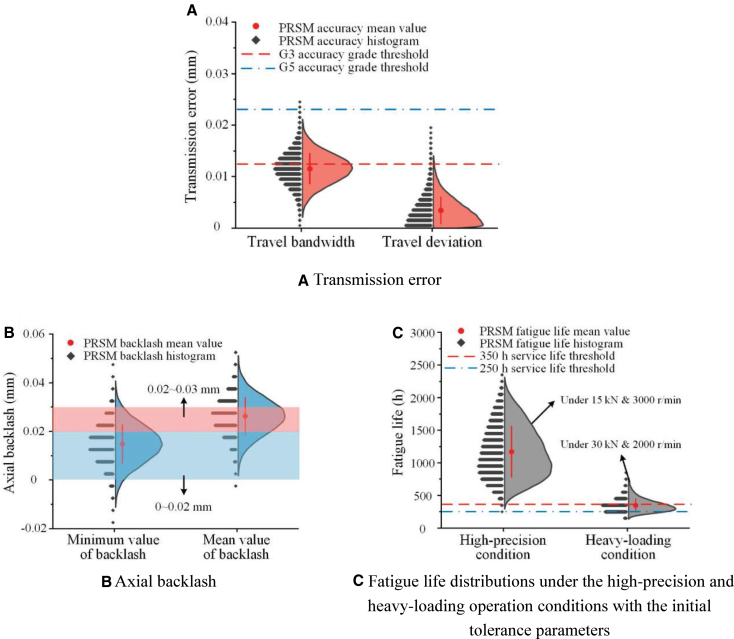
Transmission accuracy of the PRSM considering internal micro-clearances^69^ (A) Transmission error. (B) Axial backlash. (C) Fatigue life distributions under the high-precision and heavy-loading operation conditions with the initial tolerance parameters.

Thread modification significantly influences the noise, vibration, and harshness characteristics of the PRSM. By removing material from the thread flanks to achieve a uniform static load, this process alters local contact stiffness and introduces specific clearances between the mating threads. During high-speed operation, these variations in stiffness and clearance induce micro-impacts as the rollers continuously enter and exit the meshing zone. Consequently, while modification successfully improves the static load distribution, it may inadvertently amplify high-frequency vibration and acoustic noise.^67^ This reduction in dynamic smoothness is particularly critical for humanoid robots, where excessive noise degrades the human-robot interaction experience and micro-vibrations interfere with joint sensor feedback to compromise motion control precision.^68^ Therefore, the design of these modifications must carefully balance static load capacity with dynamic contact stiffness to ensure optimal performance in noise, vibration, and harshness. To address these stability issues, Liu et al.^69^ established a comprehensive evaluation model for the PRSM that accounts for axial backlash. Their study quantified the negative effects of small internal clearances on transmission accuracy, confirming that physical clearance is a primary factor driving internal micro-impacts and transmission errors, as shown in Figure 26. Furthermore, Wensing et al.^70^ demonstrated that non-linear clearance and friction significantly reduce the mechanical transparency of robotic actuators. These dynamic impacts not only excite high-frequency noise in joint sensor signals but also limit the closed-loop bandwidth and servo performance of the force control system. Together, these studies indicate that future modification designs must treat clearance control and dynamic noise, vibration, and harshness characteristics as core performance indicators.

### Measurement methods and tolerance robustness of modified PRSM

**Figure 27:**
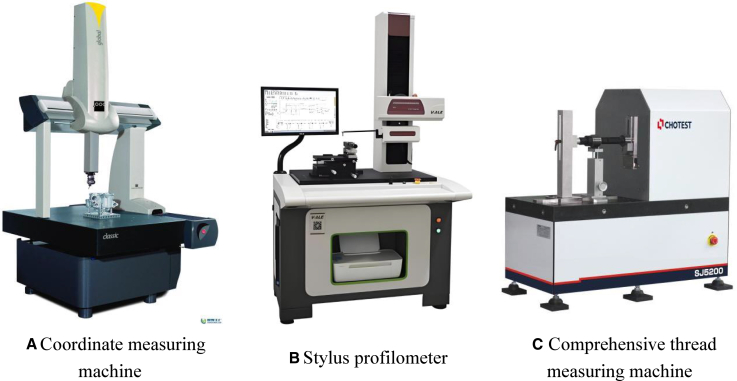
Contact measurement equipment (A) Coordinate measuring machine. (B) Stylus profilometer. (C) Comprehensive thread measuring machine.

Contact measurement is the method currently used in PRSM industrial inspection. Typical equipment includes coordinate measuring machines and stylus profilometers, as shown in Figure 27. This method gets geometric data through a physical probe touching the thread surface. It has good measurement stability and is not affected by metal surface gloss or residual machining liquid.^71^ However, traditional contact measurement methods face difficulties when measuring high-precision modified threads. Because the PRSM thread space is usually very narrow, a physical probe with a fixed radius cannot easily reach the deep thread root area, which leads to incomplete data about the root shape. Furthermore, the physical tracking process might scratch the finely modified surface, and the measurement efficiency is relatively low when dealing with complex modified surfaces.

**Figure 28:**
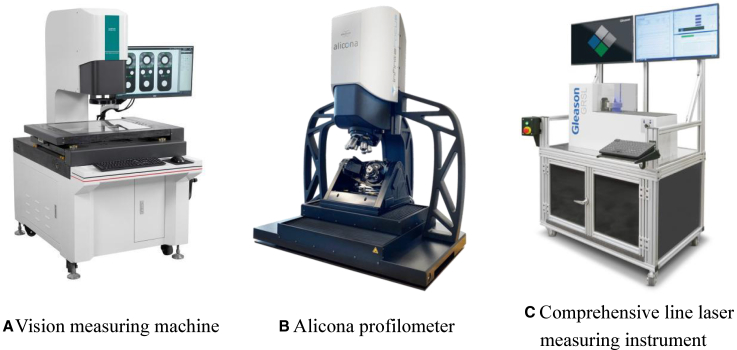
Non-contact measurement equipment (A) Vision measuring machine. (B) Alicona profilometer. (C) Comprehensive line laser measuring instrument.

In recent years, non-contact measurement has received increasing attention, mainly including machine vision, laser scanning, and structured light projection technologies, as illustrated in Figure 28. This method employs optical sensors to capture surface signals and convert them into images or high-density 3D point cloud data,^72^ with the key advantages of high measurement efficiency and a non-contact design that completely avoids damage to the modified surface. Nevertheless, non-contact measurement still presents certain limitations in practical applications: high-precision machined metal surfaces tend to induce diffuse reflection or multipath interference, leading to noise in optical data, while its accuracy in extracting complex boundary features is occasionally restricted by sensor resolution.

Beyond precise measurement, the actual application of modification design depends on its robustness to manufacturing tolerances. Because the expected modification depth is usually only a few micrometers, it is often on the same scale as standard machining errors. If the modification design is too sensitive, normal machining fluctuations will change the micro-surface or cause the threads to be over-cut, which cancels out the load-sharing benefits. A robust modification design must ensure that the stress distribution stays reasonably even, even at the maximum allowed manufacturing tolerances, and that serious stress concentration does not reappear. Including tolerance analysis in the design stage ensures that the theoretical benefits of modification are reliably turned into actual performance under standard manufacturing conditions.

Scholars have already conducted in-depth research into the measurement and manufacturing challenges mentioned above. In the field of contact measurement, Peti et al.^73^ investigated the influence of coordinate measuring machine probe size on the scanning accuracy of internal threads, confirming that traditional fixed-size probes exhibit inherent physical interference and measurement blind spots when accessing narrow and deep thread root regions. To overcome this physical limitation, Miao et al.^72^ proposed a high-precision non-contact measurement method based on machine vision for thread profile detection. By optimizing optical algorithms, this method avoids physical scratches and enables faster and more accurate inspection of complex thread surfaces. With respect to tolerance robustness, Hu et al.^74^ systematically analyzed the impact of manufacturing errors on load distribution within the PRSM, and their work revealed that even micrometer-scale manufacturing errors can break the synchronization of the system, resulting in non-uniform load distribution and a sharp rise in contact stress.

### Engineering applicability and practical considerations

From an industrial perspective, the selection of a modification strategy depends on the trade-off between performance gain and manufacturing constraints, as shown in Table 3. For instance, half-thread thickness and pitch modification exhibit high operability as they can be implemented through Computer Numerical Control (CNC) program adjustments without specialized tooling, making them cost-effective for mass-produced industrial actuators. In contrast, concave-convex arc modification requires high-precision grinding wheel dressing and complex measurement techniques, resulting in significantly higher costs. This method is typically reserved for aerospace or military applications where extreme load capacity is prioritized over cost. Furthermore, the choice of materials (e.g., GCr15 for general use vs. stainless steel for corrosive environments) influences the modification effectiveness; harder materials require higher precision in tip relief to prevent edge chipping. Operating environments also play a role; for high-speed humanoid robot joints, tip and root modification are essential to mitigate dynamic impacts and heat generation.

**Table 3:** Comparison of different thread profile modification methods for engineering applications

Modification method	Operability (manufacturing)	Relative cost	Typical materials	Working environment	Practical effect
Half-thread thickness	High (CNC grinding)	Low	GCr15, 9Cr18	Heavy load, standard temp	Excellent axial load uniformity
Pitch/lead modification	High (CNC programming)	Low	High-strength steel	High precision, long stroke	Reduces cumulative pitch error
Tooth tip/root relief	Medium (special wheel)	Medium	Carburized steel	High speed, vibration-sensitive	Reduces impact and noise
Concave-convex arc	Low (precision dressing)	High	Aerospace alloys	Extreme stress, high vacuum	Maximum reduction in contact stress

## Challenges and future works

Significant progress has been achieved in reducing first-tooth contact stress, achieving load uniformity, and enhancing the load-bearing capacity and transmission performance of PRSMs through the evolution of thread profile modification methods. However, these techniques still face diverse limitations and challenges when confronted with practical engineering applications and complex operating conditions. Therefore, it is necessary to systematically summarize the critical issues encountered in the engineering implementation of current thread teeth modification methods, and on this basis, look ahead to the future research directions and development trends. Future research efforts should focus on dynamic load analysis, high-precision modeling, the effects of machining and vibration noise, as well as rigorous experimental validation. These focal points aim to provide a robust theoretical foundation and practical guidance for the further optimization of thread modification designs.

### Challenges

First, most existing studies are conducted based on static loading conditions. However, in practical operation, PRSMs are usually subjected to complex combinations of dynamic loads, impact loads, and multi-operating conditions. Under these circumstances, the load transfer paths, contact states, and deformation characteristics may differ significantly from those under static conditions. Therefore, existing static analysis methods may have limitations in evaluating thread teeth load distribution, contact stress, and structural response. There is an urgent need to integrate dynamic analysis and multi-condition simulation techniques. Such advancements will enable a more accurate prediction of PRSM performance under actual operating conditions.

Second, high-precision modeling is a key challenge. PRSM systems involve complex multi-body meshing contact, micro-scale clearances, and coupled rolling and sliding behaviors of rollers. Furthermore, the nonlinear elastoplastic characteristics of the materials add levels of complexity to finite element modeling and numerical simulations. In the process of high-precision modeling, how to strike a balance between ensuring computational accuracy and controlling computational costs, as well as achieve appropriate mesh division and realistic boundary condition setting, remains a critical challenge that needs to be urgently addressed.

Third, current modification design strategies often overlook practical factors such as machining errors, vibration, and noise. These factors can exert a pronounced influence on the contact status and load distribution of thread teeth during high-speed operation or under high-precision loads. Consequently, theoretical optimization schemes may fail to realize their intended effectiveness in real-world engineering scenarios.

Fourth, in practical engineering applications, although thread modification can theoretically significantly improve the load distribution of the PRSM, there remain application bottlenecks in precision grinding processes. High-order modifications are highly sensitive to manufacturing errors, and micron-level tolerance deviations may cause the theoretical advantages of uniform load distribution to be lost. In addition, mature interfaces are lacking between modification algorithms and the control systems of precision grinding machines, which makes it difficult to convert theoretical models into actual grinding wheel paths. Future research should focus on robust optimization designs to improve the error tolerance of modification schemes. Meanwhile, efforts should be dedicated to developing digital interfaces that integrate design and manufacturing. By converting mathematical modification models into usable computer numerical control code, the value of modification technology in guiding engineering practice can be fully realized.

Finally, experimental verification methods remain insufficient. Presently, there is a lack of systematic, repeatable test platforms that can cover diverse loads and operating conditions, making it difficult to verify the effects of modification under actual operating conditions. This limits the effective translation of theoretical and numerical analysis results into practical engineering applications.

### Future development

To address the aforementioned challenges, future research on PRSM thread modification must evolve toward multi-dimensionality, high precision, and enhanced engineering feasibility. First, emphasis should be placed on dynamic analysis and multi-condition research. This involves incorporating varying axial loads, heavy-duty scenarios, and complex operating conditions to comprehensively assess the influence of thread modification on load distribution, contact stress, and structural deformation. By introducing dynamic simulation and multi-physics coupling analysis methods, the performance of PRSM in actual operating environments can be predicted more accurately. This approach will guide the optimized design of modification methods under complex working conditions.

Second, high-precision modeling methods should be developed. By integrating nonlinear material models, multibody contact analysis, and high-resolution meshing strategies, the accuracy and fidelity of finite element simulations can be significantly enhanced. The modeling process should comprehensively account for the impacts of roller rolling, meshing clearances, and nonlinear material behaviors on contact status and load transfer. To achieve a better match between theoretical predictions and actual performance is crucial for providing a reliable basis for optimization design. In the context of Industry 4.0, future research should further explore the integration of digital twin technology to create dynamic, real-time replicas of the PRSM during operation. By combining high-fidelity models with sensor data, digital twins can facilitate virtual compensation for manufacturing errors and thermal deformations. This evolution from static modeling to active digital synchronization will enable more precise prediction of service life and self-optimizing modification strategies for high-end robotic and aerospace applications. Beyond these advances in digital modeling, studying the fluid-structure coupling effect during high-speed operation is also a key research frontier. Most existing research on PRSM thread profile modification uses a dry friction assumption. It fails to consider the interaction when the elastohydrodynamic lubrication film thickness and the modification size are on the same micro-scale. During high-speed operation, a fluid-structure coupling effect occurs because the lubrication film thickness matches these modification gaps. The lubricant fills the modification gaps and acts as a load transfer medium. Instead of letting the designed geometric space reduce local stress, the fluid bridges these gaps and actively transfers fluid dynamic pressure. Therefore, this local fluid pressure causes secondary elastic deformation on the thread flank. This changes the effective contact geometry and the actual stress distribution. This fluid-induced load transfer may partially or completely cancel out the expected load-sharing benefits of the geometric modification. Exploring how elastohydrodynamic lubrication induced fluid-structure coupling dynamically changes the modification profile remains essential for high-speed PRSM design.

Meanwhile, modification design should comprehensively take into account practical engineering factors including manufacturing errors, vibration, and noise to improve the implementability and engineering adaptability of optimization schemes. Practical factors being introduced during the design phase, the performance deterioration of theoretical schemes in practical applications can be effectively reduced, thus improving the reliability and lifespan of PRSMs.

Finally, establishing standardized and repeatable experimental test platforms is crucial for validating theoretical and numerical analysis results. Such platforms not only provide high-precision experimental data for verifying modification methods but also support comprehensive performance evaluations under multiple loads, diverse operating conditions, and long-term operations. This provides a rigorous scientific basis and robust data support for further optimization design.

## Conclusion

PRSMs exhibit a phenomenon of non-uniform load distribution during the transmission process, which causes the thread teeth near the loaded end to bear disproportionately high loads. This condition significantly increases the susceptibility to fatigue failure and ultimately degrades the overall transmission performance. Therefore, thread profile modification is regarded as an effective approach to achieving uniform load distribution and enhancing transmission performance. Half-thread thickness modification, tooth tip modification, and root modification primarily alleviate load concentration at the first-engaged teeth via localized geometric adjustments; while these methods feature operational simplicity, their ability to regulate the global load path remains limited. In contrast, pitch diameter modification as well as concave-convex arc profile modification optimize load distribution along with contact states through global geometric adjustment, though they entail considerable design-manufacturing complexity. Moreover, parameter optimization modification achieves a balance between localized and global performance through multi-parameter synergistic design, but it imposes high demands on computational accuracy and optimization costs. Comparative analysis indicates that these varied modification methods are inherently complementary; their rational integration enables uniform load distribution across all thread teeth as well as overall performance enhancement of the system. To achieve this integration, this study proposes a systematic macro-*meso*-micro cross-scale collaborative modification framework. Within this framework, the macro scale focuses on global modification techniques, such as pitch diameter modification, global reconstruction of concave-convex arc profiles, and multi-objective parameter optimization, to adjust the load distribution trends along the meshing length, eliminate axial load concentration, and control the load across all teeth. The meso scale centers on local modification techniques like half-thread thickness modification, tooth tip modification, and root modification. By making local geometric adjustments, these methods redistribute loads between thread teeth and optimize structural strength. The micro scale focuses on optimizing the contact state of the tooth surface under actual operating conditions. This framework establishes clear links between these scales; for instance, the friction state on the micro surface affects the contact performance of the meso-scale tooth profile. In turn, the force conditions of the meso-scale tooth profile determine local performance, while the macro parameters regulate the overall load balance. Through these connections, the framework successfully combines overall load distribution with local performance.

Although existing research has achieved substantial progress, the thread profile modification of PRSMs still faces numerous challenges. There is insufficient performance evaluation of modified thread teeth under dynamic loads and complex operating conditions, with prominent issues in machining consistency and implementability. The impact of friction and wear effects on long-term service performance has not been fully considered, and standardized experimental verification is lacking. To a certain extent, these issues have restricted the popularization and optimization of modification methods in engineering applications.

Future research should prioritize multi-condition dynamic analysis as well as the systematic modeling of modified thread machining issues and friction-wear behaviors. Meanwhile, standardized experimental platforms should be established to validate theoretical and numerical analysis results, and the integration of multi-objective optimization algorithms will achieve a closed loop from theoretical analysis to design optimization. Through these approaches, it will be possible to drive the transition of thread profile modification methods from empirical designs to more comprehensive and practical design paradigms. This progression will provide a solid theoretical foundation and practical reference for the engineering application of PRSMs in high-precision, high-reliability, and complex operating conditions.

## Acknowledgments

This research was supported by the State Key Laboratory of High-performance Precision Manufacturing (grant no. HPMKF202513), major special project for technological research and development during the 14th five-year-plan period in Zhenhai district (grant no. 2024005), Key R&D program project of Science and Technology Innovation Yongjiang 2035 in Ningbo (grant no. 2025Z005), 10.13039/501100007129Shandong Provincial Natural Science Foundation (grant no. ZR2023QE314).

## Declaration of interests

The authors declare no competing interests.
